# Practical Evaluation of Standard 3D Gaussian Splatting for Real-World Scene Reconstruction

**DOI:** 10.3390/s26185754

**Published:** 2026-09-10

**Authors:** Tomohiro Mizoguchi

**Affiliations:** Department of Informatics and Data Science, Faculty of Engineering, Sanyo-Onoda City University, Sanyo-Onoda 756-0884, Yamaguchi, Japan; mizoguchi@rs.socu.ac.jp

**Keywords:** 3D Gaussian Splatting, spatial reconstruction, digital twin, photogrammetry, infrastructure inspection, 3D scene sensing

## Abstract

3D Gaussian Splatting (3DGS) enables real-time rendering of photorealistic scene representations from multiview images and has potential as a visualization layer for digital twins. However, photorealistic appearance and image-level metrics alone do not establish practical suitability. We evaluate standard 3DGS on six real-world datasets: wall, forest, piled-pier underside, steel-girder bridge, asphalt pavement, and outdoor sculpture. The framework combines held-out test-view metrics (PSNR, SSIM, and LPIPS), spatial error maps, region-of-interest (ROI) inspection, computational records, scale–opacity grouping, and Gaussian-to-MVS distance analysis. In the wall case study, extending adaptive density control from 7000 to 30,000 iterations nearly doubled Gaussian count and model size and increased training time by about 50%, with only modest test-view improvements. Scale–opacity classes did not reliably distinguish high- from low-error regions. Gaussian-to-MVS distance was more strongly associated with scale than learned opacity, and median local errors generally increased with distance, although distributions overlapped substantially. Across datasets, major structures were generally reproduced well, whereas sparse foliage, low-contrast repetitive textures, fine surface details, and distant background objects required ROI-level verification. The best test-view performance reached 37.11 dB PSNR, 0.952 SSIM, and 0.193 LPIPS. Practical evaluation should consider global quality together with local, computational, and geometric characteristics.

## 1. Introduction

In recent years, the convergence of sensing, IoT-enabled connectivity, cloud computing, and artificial intelligence (AI) has accelerated the development of Digital Twins, which maintain up-to-date representations of physical spaces to support monitoring and data-driven decision-making. In the construction and infrastructure sectors, Digital Twins are expected to support life-cycle management by linking information on existing conditions with BIM/CIM, sensor observations, and inspection and maintenance records [1,2,3,4]. Because existing structures and natural environments continuously evolve through deterioration, human intervention, and environmental change, efficient methods are needed to capture and update both the three-dimensional (3D) geometry and visual appearance of target objects.

For 3D measurement of physical spaces, LiDAR-based technologies such as Terrestrial Laser Scanning (TLS) and Mobile Laser Scanning (MLS) are widely used. LiDAR is suitable for acquiring dense and accurate point clouds; however, it can involve burdens related to equipment cost, setup and operation, measurements from multiple stations, and handling occluded areas [5,6,7]. In contrast, image-based 3D reconstruction based on Structure-from-Motion and Multi-View Stereo (SfM–MVS) can use ordinary digital cameras and UAV-mounted cameras, allowing flexible data acquisition according to field conditions [8,9,10]. Colored point clouds and textured meshes obtained by SfM–MVS can be widely used for geometric recording, measurement, and visualization of existing conditions from arbitrary viewpoints. However, these are primarily geometry-based representations in which image information is assigned to reconstructed surface geometry.

In recent years, neural rendering techniques have emerged as an alternative to explicit surface meshes. These techniques optimize scene appearance from multi-view images and directly synthesize novel-view images. Neural Radiance Fields (NeRF) achieved high-quality novel-view synthesis, but training and rendering are computationally expensive because they require numerous ray samples and network evaluations [11]. In contrast, 3D Gaussian Splatting (3DGS), proposed by Kerbl et al., explicitly represents a scene as a set of numerous anisotropic 3D Gaussian primitives and enables fast optimization and real-time rendering [12]. Unlike conventional textured meshes, 3DGS directly optimizes scene appearance from multi-view images and represents view-dependent color using spherical harmonics, enabling smoother and more photorealistic novel-view rendering. Its application to the visualization of existing buildings, urban environments, infrastructure facilities, and natural environments has therefore been increasingly explored [13,14,15].

Despite its high visual fidelity, 3DGS does not necessarily ensure geometric completeness or faithful reproduction of fine details. This is because 3DGS is a radiance-field representation that optimizes Gaussian primitives to minimize multi-view image-reproduction error, rather than a geometric model that directly represents object surfaces as point clouds or meshes do. Therefore, novel-view rendering quality and the spatial correspondence of the Gaussian distribution with physical target surfaces should be evaluated from different perspectives. In particular, when 3DGS is combined with point clouds, BIM/CIM, inspection records, or maintenance information in Digital Twin and inspection-support applications, it is necessary to examine not only photorealistic appearance, but also the visibility of target regions of interest (ROIs), computational feasibility, and relative consistency with reference geometry. Because MVS geometry is itself reconstructed from multi-view images, it may contain errors arising from image matching, camera-pose estimation, triangulation, and dense stereo reconstruction. Therefore, Gaussian-to-MVS distances should be interpreted as relative consistency with an image-based reconstruction rather than as absolute geometric accuracy.

Among these evaluation aspects, local visibility is particularly important in inspection and maintenance applications. Features such as cracks, corrosion, leakage traces, and coating damage often occupy only a small portion of an image but can be important for condition assessment. However, widely used image-quality and perceptual-similarity metrics, such as Peak Signal-to-Noise Ratio (PSNR), Structural Similarity Index Measure (SSIM), and Learned Perceptual Image Patch Similarity (LPIPS), are typically reported as single values for an entire image. Consequently, blurring or omission of small local features may not be strongly reflected in these metrics. Local artifacts may appear as blurring, color bleeding, texture smoothing, or indistinct boundaries. These artifacts are particularly likely to occur around thin structures, small defects, object boundaries, depth discontinuities, occluded regions, poorly illuminated areas, and low-contrast or repetitive textures. Therefore, even when the overall rendering appears visually plausible, fine details required for inspection may remain unclear. Practical evaluation should therefore consider not only global image-quality metrics, but also the spatial distribution of errors and the visibility of target ROIs.

Based on these considerations, this study clarifies the evaluation aspects that should be combined when standard 3DGS is used to represent existing real-world environments as a visualization layer for Digital Twins and inspection support. The central claim of this study is that practical use of standard 3DGS requires an integrated evaluation that considers not only global rendering quality based on PSNR, SSIM, and LPIPS, but also local image-space errors; visibility of target ROIs; computational load based on the number of Gaussians, VRAM usage, training time, and model size; internal Gaussian attributes such as scale and opacity; and relative geometric consistency with MVS reference geometry.

Accordingly, this study evaluates standard 3DGS across six real-world image sequences using an integrated assessment of rendering quality, local errors, computational cost, Gaussian attributes, and MVS-relative geometry. The specific research questions and contributions are presented in Section 2.3.

## 2. Related Work and Research Objective

### 2.1. Real-World Scene Reconstruction

3DGS can achieve both high-quality novel-view synthesis and fast rendering. Accordingly, its application to 3D recording and visualization of real-world environments has been increasingly explored in fields such as construction and infrastructure, photogrammetry, remote sensing, cultural heritage documentation, and forest and vegetation monitoring.

For buildings and urban environments, several studies have examined the construction of 3DGS models from terrestrial or UAV images. Cai et al. [13] applied 3DGS to building reconstruction and demonstrated its potential for visual representation of real-world environments. Atik [16] compared point clouds generated using NeRF- and 3DGS-based methods from UAV imagery with photogrammetric point clouds obtained using SfM–MVS, using rendering quality, M3C2 distance, and cross-sectional analysis. Fadilah et al. [17] evaluated multiple 3DGS implementations for UAV-based urban heritage reconstruction using rendering metrics and M3C2 distances to MVS point clouds. Their results indicated that rendering quality and geometric accuracy do not necessarily correspond, suggesting that these aspects should be evaluated separately in real-world applications.

Research has also addressed larger-scale urban environments and georeferencing. Yan et al. [18] compared multiple 3DGS methods for urban-scene reconstruction and evaluated geometric accuracy, rendering quality, scalability, and computational efficiency using photogrammetric and LiDAR-derived reference data. Their results showed trade-offs among geometric accuracy, photorealistic rendering quality, and computational resource consumption. Hou et al. [14] proposed GeoRefGS for UAV image-based 3DGS by introducing a learnable similarity transformation and georeferencing constraints that connect a local 3DGS coordinate system with a global geospatial coordinate system. For large-scale scenes, VastGaussian [19] divides a scene into multiple regions, optimizes them separately, and subsequently integrates the results. These studies show that georeferencing, scene partitioning, computational requirements, appearance variation, and integration artifacts are important considerations in large-scale applications.

In forests and vegetation environments, reconstruction challenges differ from those in artificial structures because of complex tree-crown geometry, variations in foliage density, self-occlusion, and limited visibility of trunks and ground surfaces. Shaheen et al. [20] proposed ForestSplat, which combines consumer-grade UAV imagery, SfM–MVS, and 3DGS, and demonstrated its applicability to large-scale forest mapping and canopy-height estimation through comparison with airborne LiDAR data. Tian et al. [21] compared 3DGS, NeRF, and conventional photogrammetry for urban forest reconstruction and evaluated the extraction of tree height, diameter at breast height, and crown diameter using TLS point clouds as reference data. Petrovska and Jutzi [15] compared the effects of vegetation occlusion on MVS, NeRF, 3DGS, and 2DGS and analyzed differences in reconstruction completeness and accuracy under occlusion.

### 2.2. Densification, Efficiency, and Reconstruction Characteristics

In 3DGS, the Gaussian set is dynamically updated during training through densification and pruning. Densification duplicates or splits Gaussians in regions where the current representation is insufficient, based on criteria such as the view-space positional gradient. Such regions often include object boundaries, fine geometric details, and high-frequency textures. However, increasing the number of Gaussians also increases VRAM usage, training time, and model size. Practical applications therefore require evaluation of both image-quality improvement and computational cost.

Several methods have been proposed to improve computational efficiency and reduce model size. Papantonakis et al. [22] reduced the memory footprint of 3DGS representations by combining resolution-aware Gaussian pruning, adaptive reduction in spherical harmonics coefficients, and attribute quantization. Mallick et al. [23] introduced budget-constrained densification and score-based sampling to construct high-quality radiance fields while controlling Gaussian count, model size, and training time.

Continued densification does not necessarily improve local reconstruction quality uniformly. Ye et al. [24] showed that gradient collision, in which gradients from different viewpoints or pixels cancel each other, can prevent large Gaussians from being sufficiently split, leaving missing details and blur. FreGS [25] used differences in the frequency spectra of rendered and reference images and progressively regularized the model from coarse, low-frequency structures to fine, high-frequency details.

Image-quality metrics aggregated over an entire image, such as PSNR, SSIM, and LPIPS, are widely used to evaluate 3DGS [26,27,28]. Fang et al. [29] examined how the number of images, number of iterations, input resolution, and densification-related parameters affect rendering quality and processing time in indoor-scene generation. Martin et al. [30] presented a subjective quality-assessment benchmark for Gaussian Splatting and analyzed the correspondence between objective metrics and human perception. Their results showed that artifacts such as floaters and inconsistencies in occluded regions may be visually apparent but insufficiently reflected in image-level metrics. Therefore, global metrics should be complemented by spatially resolved assessments using error maps and enlarged ROI inspections.

Standard 3DGS is designed for image reproduction, and individual Gaussians do not directly represent physical surfaces in the same manner as point clouds or meshes. Previous studies have shown that rendering quality and geometric agreement with reference data do not necessarily correlate [17,18]. Image-space appearance and spatial consistency with MVS- or LiDAR-derived reference geometry should therefore be treated as distinct evaluation aspects.

However, few studies have jointly applied these evaluation aspects to multiple real-world image sequences using a common standard 3DGS pipeline. The relationships among rendering quality, local errors, computational cost, Gaussian attributes, and reference geometry therefore remain insufficiently organized for practical use.

### 2.3. Research Objective

The objective of this study is to establish the evaluation aspects needed to assess the practical suitability of standard 3DGS as a potential visualization layer for future Digital Twin and inspection-support workflows. Rather than proposing a new 3DGS algorithm, this study evaluates its reconstruction characteristics under practical image-acquisition conditions from the perspectives of image quality, local errors, computational load, Gaussian attributes, and spatial correspondence with reference geometry.

Based on this objective, this study addresses the following research questions.

RQ 1: Under a fixed 30,000-iteration optimization budget, how does the duration of adaptive density control affect held-out test-view quality, local error distributions, Gaussian count, VRAM usage, training time, and model size? (Section 4.)
RQ 2: To what extent can simple internal Gaussian attributes, such as scale and learned opacity, provide auxiliary information about the spatial distribution of local image errors? (Section 5.)
RQ 3: How is Gaussian-to-MVS center distance distributed relative to the MVS reference surface, and how does it relate to Gaussian attributes and projected local errors? (Section 6.)
RQ 4: Under which scene and acquisition conditions does standard 3DGS yield stable reconstructions, and where do local artifacts persist? (Section 7.)

The main contributions of this study are as follows: (1) a structured practical evaluation procedure for examining standard 3DGS from the perspectives of rendering quality, local errors, computational requirements, Gaussian attributes, and MVS-relative geometry; (2) a quantitative comparison of the quality gains and computational costs associated with extending adaptive density control; (3) an exploratory assessment of the associations between simple Gaussian attributes and local image-space errors; (4) an assessment of Gaussian-center proximity to an MVS-reconstructed shape and its relationship with image-space errors; and (5) a cross-scene synthesis of the conditions under which reconstruction was stable or local artifacts remained across six real-world datasets.

The practical evaluation framework presented in this study is not intended to replace geometric accuracy verification based on independent surveying references. Rather, it is positioned as a structured evaluation procedure before practical use, designed to examine whether inspection-relevant details remain sufficiently visible in target ROIs, whether the model can be constructed and operated within the available computational environment, and whether major spatial inconsistencies are apparent relative to image-based reference geometry.

The answers to these research questions are summarized explicitly in Section 8.4.

## 3. Materials and Methods

**Figure 1:**
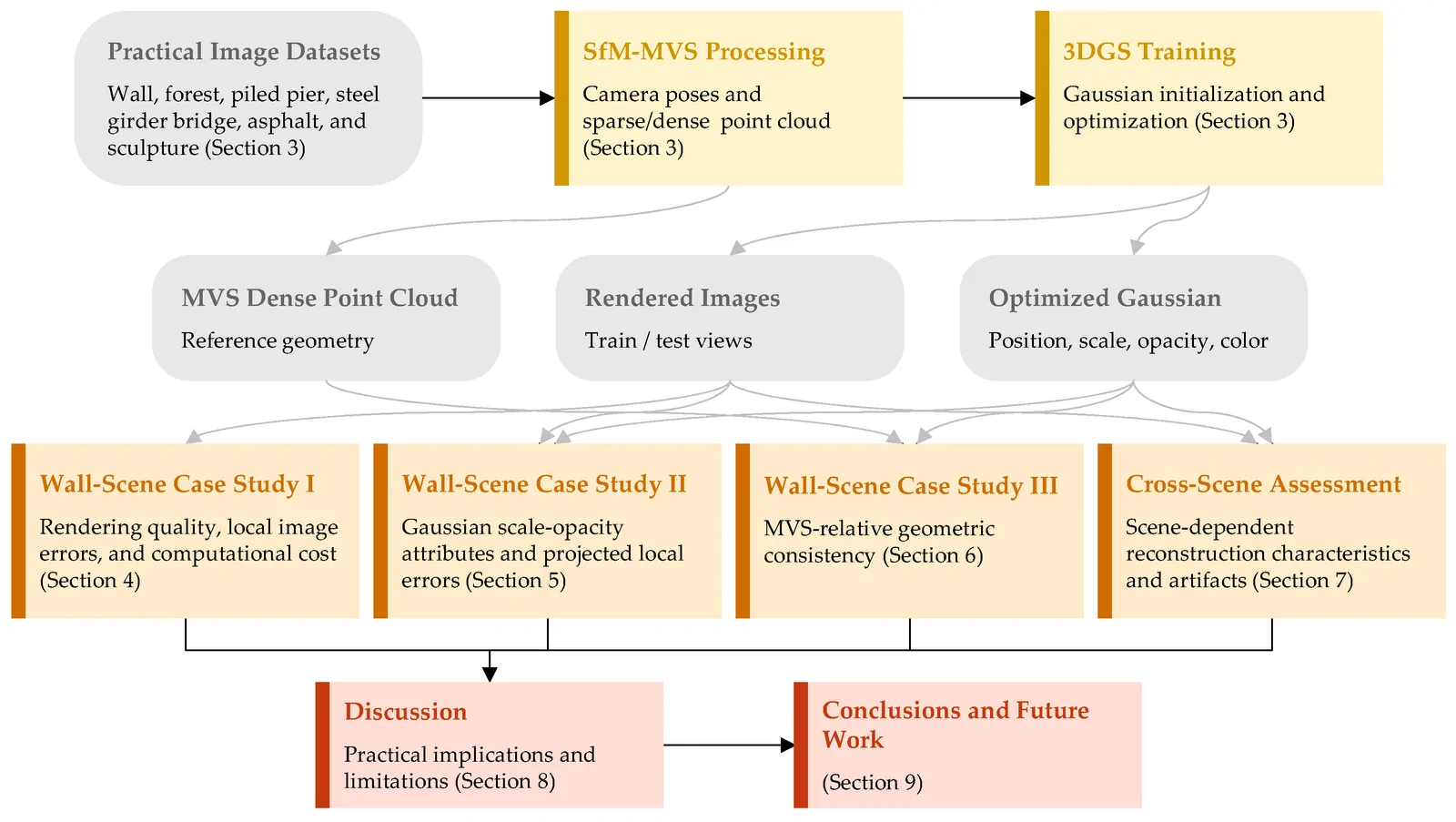
Practical diagnostic evaluation framework for standard 3DGS in real-world 3D scene sensing. Practical image datasets were processed using Structure-from-Motion and Multi-View Stereo (SfM–MVS) and standard 3D Gaussian Splatting (3DGS). The resulting Multi-View Stereo (MVS) point clouds, rendered images, optimized Gaussian attributes, and computational records were jointly used to evaluate rendering quality, local errors, Gaussian-level diagnostics, computational burden, and MVS-relative geometric consistency. The colors are used only to visually distinguish the workflow components, and the arrows indicate the processing and evaluation flow.

Figure 1 presents an overview of the diagnostic evaluation framework adopted in this study and summarizes the correspondence between its evaluation components and the analyses presented in the subsequent sections.

### 3.1. Overview of 3D Gaussian Splatting

3DGS explicitly represents a scene as a collection of numerous anisotropic 3D Gaussian primitives and generates novel-view images by projecting these primitives onto the image plane and compositing them using alpha blending [12]. A sparse 3D point cloud estimated by SfM is typically used to initialize the Gaussian positions, after which the position, shape, opacity, and color of each Gaussian are updated through optimization. Each Gaussian $G_{i}$ is represented by a center position $\mu_{i}\in R^{3}$, a covariance matrix $\Sigma_{i}\in R^{3\times 3}$, a learned opacity, and spherical harmonics (SH) coefficients that represent view-dependent color. For a position $x\in R^{3}$, the Gaussian is expressed as

(1) $$G_{i}\left(x\right)=exp\left(-\frac{1}{2}{\left(x-\mu_{i}\right)}^{T}\Sigma_{i}^{-1}\left(x-\mu_{i}\right)\right).$$

To preserve the positive definiteness of the covariance matrix and enable stable optimization, $\Sigma_{i}$ is parameterized using a scale matrix $S_{i}$ and a rotation matrix $R_{i}$ as

(2) $$\Sigma_{i}=R_{i}S_{i}S_{i}^{T}R_{i}^{T}.$$

During rendering, each 3D Gaussian is transformed into the camera coordinate system and projected onto the image plane. Let W denote the transformation into camera coordinates and $J_{i}$ denote the Jacobian of the local affine approximation of the projection. The projected two-dimensional covariance matrix $\Sigma_{i}^{\prime }$ is then approximated as

(3) $$\Sigma_{i}^{\prime }=J_{i}W\Sigma_{i}W^{T}J_{i}^{T}$$

Thus, an anisotropic Gaussian in 3D space is rendered as an elliptical two-dimensional Gaussian on the image plane. The color of each Gaussian is obtained by evaluating its SH coefficients according to the viewing direction. The color of a pixel p is calculated by sorting the Gaussians that contribute to that pixel in depth order and applying front-to-back alpha compositing. Here, $\alpha_{i}(p)$ denotes the effective alpha value of Gaussian i at pixel p. It is determined by both the learned opacity stored by the Gaussian and the weight assigned to pixel p by the projected two-dimensional Gaussian. Therefore, $\alpha_{i}(p)$ is not the Gaussian’s learned opacity alone. It is the effective opacity at pixel p, which also depends on the Gaussian’s projected position and shape. Let N denote the number of Gaussians contributing to pixel p, and $c_{i}$ the view-dependent color of Gaussian i. The pixel color C(p) is then given by

(4) $$C(p)=\sum_{i=1}^{N}{c_{i}\alpha }_{i}(p)\prod_{j=1}^{i-1}\left(1-\alpha_{j}\left(p\right)\right)$$

During training, the parameters of each Gaussian are optimized by gradient descent so as to minimize the difference between the rendered image and the input image. Standard 3DGS uses a loss function that combines an $L_{1}$ loss and a D-SSIM loss:

(5) $$L=\left(1-\lambda \right)L_{1}+\lambda L_{D-SSIM}$$

In addition, the number of Gaussians is not fixed during training because adaptive density control is applied. Specifically, densification duplicates or splits Gaussians based on criteria such as the view-space positional gradient, whereas Gaussians with low opacity or excessively large scale are removed through pruning. Opacity reset is also performed at specified intervals, thereby affecting the opacity distribution and subsequent pruning behavior. Consequently, the number, scale, opacity, and spatial distribution of Gaussians change dynamically as training progresses.

### 3.2. Target Scenes and Image Acquisition

This study used real-world image sequences of a wall, forest, piled-pier underside, steel-girder bridge, asphalt pavement, and outdoor sculpture. The main acquisition conditions for each scene are summarized in Table 1. The wall, asphalt pavement, and sculpture datasets were newly acquired for this study, whereas the other image sequences had previously been collected for practical purposes. Because the camera type, image resolution, number of images, shooting distance, camera trajectory, and target extent differed among the scenes, the cross-scene analysis in Section 7 was treated as a descriptive assessment rather than as a controlled comparison isolating individual acquisition factors.

The wall scene, whose primary target was an approximately planar masonry wall, was selected for the detailed case study. In addition to the wall blocks and joints, the scene included a fence, soil, gravel, vegetation, sky, and a distant lighting pole. This image sequence therefore allowed relatively stable planar regions to be compared with regions containing thin structures, repetitive patterns, depth discontinuities, background regions containing the sky and distant objects, and nonuniform textures.

**Figure 2:**
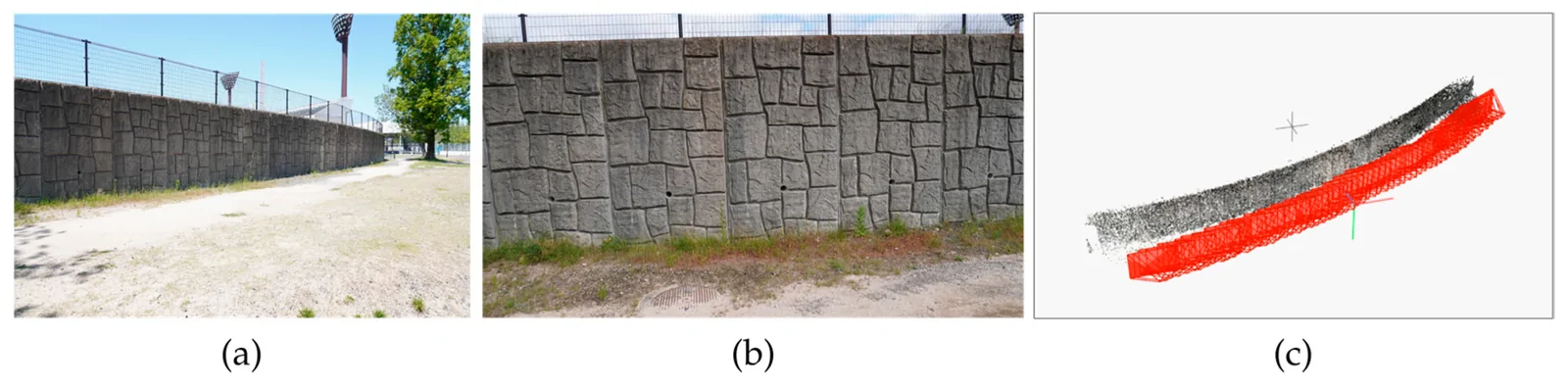
Wall-scene dataset used for the detailed analyses. (**a**) Target wall and surrounding environment; (**b**) representative input image; (**c**) SfM sparse point cloud and estimated camera poses. The three colored lines indicate the coordinate axes and are shown only for visualization.

The wall scene was captured with a handheld camera from approximately frontal viewpoints while maintaining a distance of about 5 m from the wall. A total of 67 images were acquired at 1 s intervals using interval shooting. COLMAP was used to estimate the SfM sparse point cloud and camera parameters, which were then used as the initial input for 3DGS. The original image resolution was 6000 × 3376 pixels, and the images were downsampled to 1500 × 844 pixels for 3DGS input. Figure 2 shows the target wall, a representative input image, and the SfM sparse point cloud with the estimated camera trajectory.

Following the evaluation split adopted in the official 3DGS implementation, the images were ordered according to the acquisition sequence, and every eighth image was selected as a test image; the remaining images were used for training. For the wall scene, nine test images were selected, leaving 58 images for training. For the steel-girder bridge scene, the test images were subsampled to avoid including an excessive number of closely spaced frames. For the forest, piled-pier underside, asphalt pavement, and sculpture scenes, the standard interval-based selection was applied.

The test images were excluded from 3DGS optimization. However, both the training and test images were included in the COLMAP-based SfM processing to estimate their camera poses. The evaluation therefore measured held-out test-view rendering quality at known camera poses and did not evaluate camera-pose estimation.

For video-derived image sequences, regularly sampled test viewpoints may remain close to adjacent training viewpoints because of dense temporal sampling. This was particularly relevant to the steel-girder bridge scene, which included periods of slow movement and hovering. Therefore, the PSNR, SSIM, and LPIPS values for the video-derived scenes may primarily reflect near-view interpolation within highly overlapping image sequences rather than generalization to more widely separated viewpoints.

To examine the effect of greater spatial separation between training and test viewpoints, an additional continuous hold-out experiment was conducted for the wall scene. Nine consecutive images near the middle of the acquisition sequence were reserved as test images, and the remaining 58 images were used for training. Thus, the numbers of training and test images were identical to those in the regular every-eighth split. The model was trained using the Densify-30k setting with the same image resolution, optimization parameters, and 30,000-iteration budget as in the main wall-scene experiment. As in the regular split, all 67 images were included in COLMAP processing for camera-pose estimation, whereas the nine hold-out images were excluded from 3DGS optimization.

To quantify the separation between training and test viewpoints, the Euclidean distance from each test-camera center to its nearest training-camera center was calculated after applying the scale factor described in Section 3.3. The angular difference between the viewing directions of each test camera and its nearest-position training camera was also calculated. The viewing direction was defined as the camera optical-axis unit vector derived from the estimated rotation matrix, and the angular difference was calculated from the angle between the viewing-direction vectors of each test camera and its nearest-position training camera. These quantities were compared between the regular every-eighth split and the continuous hold-out split.

### 3.3. Training Settings and Evaluation Metrics

This section summarizes the training configuration, rendering-quality and spatial-error metrics, computational records, approximate real-world scaling, and the bridge image-sequence-thinning experiment.

**Training and densification settings.** The official implementation of standard 3DGS was used. The maximum number of iterations was set to 30,000, the D-SSIM loss weight to 0.2, and the spherical harmonics degree to 3. Densification was performed every 100 iterations beginning at iteration 500. The opacity reset interval was set to 3000 iterations, the densification gradient threshold to 0.0002, and the background to black. Unless otherwise stated, the remaining settings followed the defaults of the official implementation. All experiments were conducted using Google Colab GPU runtimes. The forest scene was processed on an NVIDIA H100 GPU, whereas all other experiments were performed on NVIDIA A100 GPUs.

For the detailed wall-scene case study, two settings of the densify_until_iter parameter were compared: Densify-30k (densify_until_iter = 30,000) and Densify-7k (densify_until_iter = 7000). All other training hyperparameters and the train/test split were identical. In the implementation used in this study, densify_until_iter controlled the adaptive density-control block, including the accumulation of densification statistics, Gaussian densification and pruning, and periodic opacity reset, whereas gradient-based optimization continued until iteration 30,000 in both conditions. To assess run-to-run variability, the Densify-30k and Densify-7k conditions were each evaluated using three runs with different random seeds. The same training hyperparameters, input images, and train/test split were used for all runs. Final Gaussian count, PLY file size, training time, PSNR, SSIM, and LPIPS are reported as the mean ± standard deviation across the three runs in the cross-scene summary presented in Section 7.6. The detailed Gaussian-attribute and MVS-relative analyses in Section 5 and Section 6 were conducted using the original Densify-30k run.

**Rendering-quality and spatial-error metrics.** PSNR, SSIM, and LPIPS were used as the primary rendering-quality metrics. Higher PSNR and SSIM values and lower LPIPS values indicate greater similarity to the reference images. LPIPS was computed using the VGG-based implementation in lpipsPyTorch (LPIPS version 0.1), following the evaluation script provided with the official 3DGS implementation. Training progress was evaluated every 300 iterations. At each evaluation point, the metrics were calculated separately for the training and test images, and the mean value over each image set was reported.

To evaluate the spatial distribution of image-space errors, the RGB values were normalized to the range from 0 to 1, and the pixel-wise RGB mean squared error (MSE) was calculated as

(6) $$e(x,y)=\frac{{\left(R_{gt}-R_{render}{)}^{2}+(G_{gt}-G_{render}{)}^{2}+(B_{gt}-B_{render}\right)}^{2}}{3}$$

Here, *R*, *G*, and *B* denote the color components at each pixel. The pixel-wise RGB MSE map was used to visualize the spatial distribution of pixel-value errors. The SSIM map was computed from the normalized RGB reference and rendered images using an 11 × 11-pixel Gaussian window with a standard deviation of 1.5, a data range of 1.0, and population covariance normalization. The local SSIM error map was defined as 1-SSIM.

**ROI-level and target-focused quantitative evaluation.** Representative ROIs were defined for the forest, piled-pier underside, steel-girder bridge, asphalt pavement, and sculpture scenes to examine the visibility and reconstruction quality of local scene features. The selected ROIs represented characteristic local structures or textures, including vegetation crowns, structural details, surface deterioration or discoloration, pavement textures, object boundaries, and fine surface features. For each ROI, the same ROI location and dimensions were applied to the reference image and the corresponding rendered image. PSNR, SSIM, and LPIPS were then calculated within each ROI using the same evaluation procedure as that used for the full-image assessment. The ROI locations are indicated by colored rectangles in the corresponding reference images presented in the cross-scene assessment in Section 7. The ROI sizes and quantitative results are reported with the cross-scene assessment in Section 7. The ROI analysis was intended to complement the whole-image metrics by assessing local reconstruction quality at representative target features rather than to provide an unbiased statistical sampling of all image regions.

**Target-focused quantitative evaluation for the sculpture scene.** In addition to the ROI-level assessment, a target-focused quantitative evaluation was performed for the sculpture scene using fixed foreground bounding boxes. Two representative test views were selected, and in each view a rectangular region enclosing the primary sculpture target was defined. The same bounding box was applied to the reference and rendered images, and PSNR, SSIM, and LPIPS were calculated within the corresponding region. These target-focused metrics were compared with the whole-image metrics to examine the influence of non-target background regions on scene-level evaluation.

**Computational records and approximate scaling.** The number of Gaussians, VRAM usage, cumulative training time, and final PLY file size were recorded as computational characteristics. Current allocated VRAM was used for the iteration-wise wall-scene analysis, whereas peak allocated VRAM over the full training process was used for the cross-scene comparison.

To express Gaussian scales and point-cloud distances in approximate real-world units, a uniform scale factor was calculated from the measured wall height, $H_{real}=2.6\;m$, and the corresponding distance in the SfM coordinate system, $H_{sfm}=1.1$, giving $s=H_{real}/H_{sfm}=2.36$. This factor was applied to the SfM and MVS point clouds, camera positions, Gaussian-center coordinates, and Gaussian scales. The conversion was used only for approximate interpretation and does not guarantee absolute surveying accuracy.

**Bridge image-sequence thinning.** For the steel-girder bridge thinning experiment described in Section 7.7, training sets of 200, 100, and 50 images were evaluated using the same nine test images. Each image set was processed independently using COLMAP, and all 3DGS models were trained for 30,000 iterations. Because the COLMAP reconstruction was generated separately for each image set, the experiment was interpreted as an end-to-end image-sequence-thinning comparison.

### 3.4. Gaussian Attribute and Local Error Analysis

This section examines how Gaussian scale and learned opacity are associated with local image-space errors in the wall scene. These attributes are treated as auxiliary descriptors of the internal representation rather than as direct indicators of reconstruction correctness.

**Gaussian parameter extraction.** Gaussian-center coordinates and the stored scaling and opacity parameters were extracted from the saved models at selected training iterations and from the final model. The stored scaling and opacity parameters were converted using the exponential and sigmoid activation functions, respectively. Hereafter, learned opacity refers to the activated per-Gaussian opacity value. The mean scale of each Gaussian was defined as the arithmetic mean of the three activated scale components corresponding to the diagonal entries of its scale matrix. The scale factor defined in Section 3.3 was applied to the Gaussian centers and scales. Changes in the Gaussian count and mean-scale distribution were evaluated across the saved iterations.

**Scale–opacity classification.** The Gaussians in each final model were classified according to scale and learned opacity. Let P95 denote the 95th percentile of the mean-scale distribution. Gaussians with mean scales greater than P95 were defined as large Gaussians, whereas those at or below P95 were defined as normal Gaussians. The P95 threshold was selected to represent the upper tail of the scale distribution without relying only on a small number of extreme values.

The median learned opacity in each final model was used as the opacity threshold. Values below the median were classified as low opacity, and values equal to or above the median were classified as high opacity. Each Gaussian was therefore assigned to one of four groups: large + low opacity, large + high opacity, normal + low opacity, and normal + high opacity. Up to 5000 Gaussians were randomly sampled from each group for visualization. The projected distributions were therefore used to compare the spatial locations of the groups rather than their relative population sizes.

**Projection and local-error sampling.** Gaussian centers were projected onto a representative test image using the estimated camera intrinsic and extrinsic parameters. Gaussians with non-positive depth in the camera coordinate system or projections outside the image boundaries were excluded. The projected coordinates were rounded to the nearest integer pixel.

Local MSE and local SSIM error were defined as the mean values of the pixel-wise RGB MSE map and the 1-SSIM map, respectively, within a 5 × 5-pixel neighborhood centered on each projected location. This neighborhood localized the assessment around each projected center while limiting the influence of surrounding regions. Neighborhoods intersecting the image boundary were clipped to the valid image area. Accordingly, the sampled local errors were not attributed to individual Gaussians as their rendering errors, but were used to characterize coarse spatial associations between projected Gaussian-center locations and local image-space errors.

**Interpretation of learned opacity.** The learned opacity used for grouping is a per-Gaussian parameter and does not represent the actual rendering contribution of a Gaussian in a specific view. The effective contribution depends on the projected footprint, view-dependent visibility, depth ordering, accumulated transmittance, overlap with other Gaussians, and the resulting alpha-composited weight. Because these factors were not considered, the observed relationships were interpreted as statistical associations between Gaussian attributes and local image errors. The scale–opacity grouping was therefore treated as an exploratory and auxiliary analysis rather than as a contribution-aware evaluation of individual Gaussians.

**Boxplot definition.** Unless otherwise stated, the boxplots used in the subsequent Gaussian-attribute and MVS-relative analyses were constructed using all valid samples. The boxes indicate the interquartile ranges (Q1–Q3), the center lines indicate the medians, the whiskers indicate the 10th–90th percentile ranges, and the triangular markers indicate the arithmetic means. 

### 3.5. MVS-Relative Geometric Analysis

This section evaluates the three-dimensional arrangement of Gaussian centers relative to the wall surface reconstructed by MVS.

**MVS reference geometry.** A dense MVS point cloud was generated from the wall image sequence using COLMAP. Because it was generated in the same coordinate system as the SfM reconstruction used to initialize 3DGS, it could be directly compared with the Gaussian centers extracted from the trained model. The uniform scale factor defined in Section 3.3 was applied to both the MVS point cloud and the Gaussian centers.

The original MVS reconstruction contained the ground and fence in addition to the wall. Therefore, only the wall region, which was the primary target, was manually extracted as the reference point cloud and resampled at an approximately 1 cm spacing to reduce local variations in point density.

**Gaussian–MVS distance analysis.** For each Gaussian center, the Euclidean distance to its nearest point in the wall MVS reference point cloud was calculated and defined as the Gaussian–MVS distance. To exclude Gaussians belonging to non-target regions far from the wall, only Gaussians with a Gaussian–MVS distance of 20 cm or less were included; these are hereafter referred to as wall-near Gaussians. The distance distribution was evaluated using median and percentile statistics and was compared with Gaussian scale and the four scale–opacity groups defined in Section 3.4.

**Cross-sectional and image-space analyses.** The MVS reference points and wall-near Gaussian centers were visualized in a representative cross-section to examine the spatial thickness of the Gaussian-center distribution around the reference surface. A 6 cm thick slice centered at the median y-coordinate of the MVS wall point cloud was selected, and Gaussian centers within the same y-range were extracted from the wall-near subset. The selected MVS points and Gaussian centers were projected onto the x–z plane, and a quadratic polynomial was fitted to the MVS wall profile. Signed distances from the selected Gaussian centers to the fitted curve were calculated to characterize their front-to-back distribution. The main geometric analysis, by contrast, used the unsigned three-dimensional nearest-neighbor distance.

Using the local MSE and local SSIM error values defined in Section 3.4, the wall-near Gaussians were grouped into Gaussian–MVS distance bins, and the error distributions within each bin were summarized using medians and percentile ranges.

**Scope of geometric interpretation.** The Gaussian–MVS distance was based on Gaussian centers and did not account for Gaussian scale, anisotropy, or spatial extent. In addition, the MVS point cloud was generated from the same image sequence as the 3DGS model and was therefore not an independent ground-truth reference. Accordingly, the analysis was positioned as a supplementary assessment of relative spatial consistency between the Gaussian centers and the MVS-reconstructed shape, rather than as an evaluation of absolute surveying accuracy.

## 4. Wall-Scene Case Study I: Rendering Quality and Computational Cost

### 4.1. Computational Cost Under Densification Control

**Figure 3:**
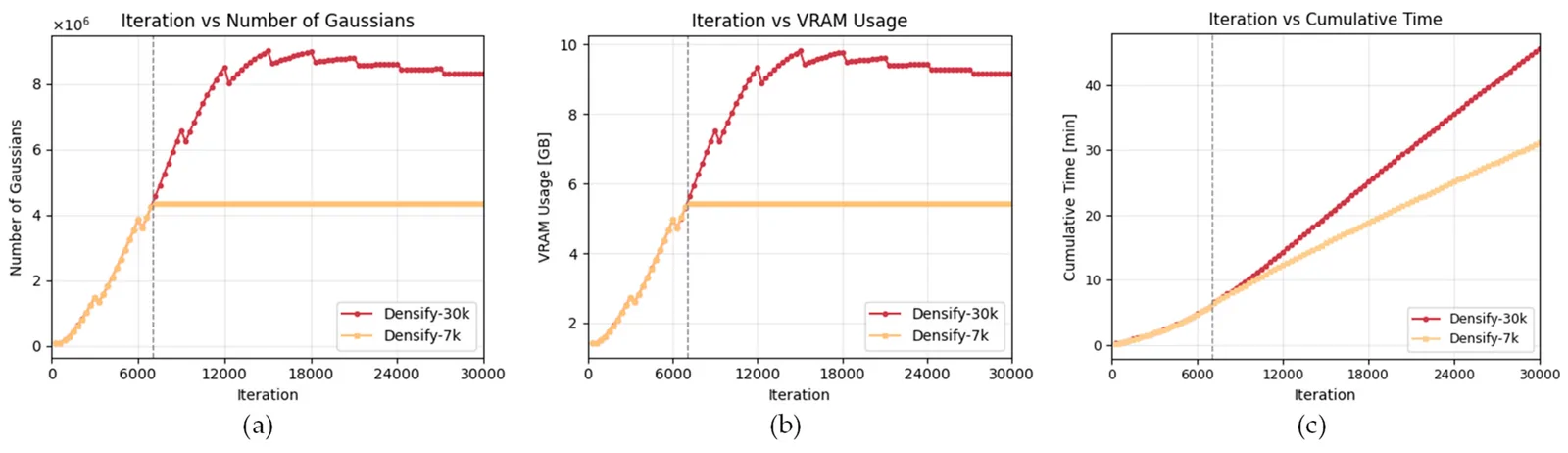
Comparison of densification behavior under the Densify-30k and Densify-7k conditions. (**a**) Number of Gaussians, (**b**) VRAM usage, and (**c**) cumulative training time as a function of iteration. The dashed vertical line indicates 7000 iterations, where densification is stopped in the Densify-7k condition. Densify-30k continues to increase the number of Gaussians and VRAM usage after 7000 iterations, whereas Densify-7k keeps both values nearly constant. In contrast, cumulative training time continues to increase in both conditions, with Densify-30k requiring a longer training time due to the continued densification process.

To evaluate the effects of continued densification on computational cost and model size, the Densify-30k condition, in which densification continued until the end of training, was compared with the Densify-7k condition, in which densification was stopped at iteration 7000. Figure 3 shows the changes in the number of Gaussians, current allocated VRAM, and cumulative processing time. The PLY file size at iteration 30,000 was also recorded.

The number of Gaussians increased rapidly during the early stage of training under both conditions. Under the Densify-7k condition, it remained nearly constant at approximately 4.4 million after densification was stopped. In contrast, under the Densify-30k condition, the number continued to increase, reaching a maximum of approximately 9.0 million at around iteration 15,000. It subsequently decreased because of pruning and reached approximately 8.3 million at iteration 30,000. The final number of Gaussians under the Densify-30k condition was approximately 1.9 times that under the Densify-7k condition.

Current allocated VRAM showed a similar trend to the number of Gaussians. Under the Densify-7k condition, it stabilized at approximately 5.5 GB, whereas under the Densify-30k condition, it reached a maximum of approximately 10.0 GB.

At iteration 30,000, the cumulative training time was approximately 33 min for the Densify-7k condition and approximately 48 min for the Densify-30k condition, indicating that the latter required approximately 1.5 times longer. The corresponding PLY file sizes were approximately 1.0 GB and 1.9 GB, respectively, representing an approximately 1.9-fold difference, which was similar to the difference in the number of Gaussians.

### 4.2. Rendering Quality and Training–Test Trends

**Figure 4:**
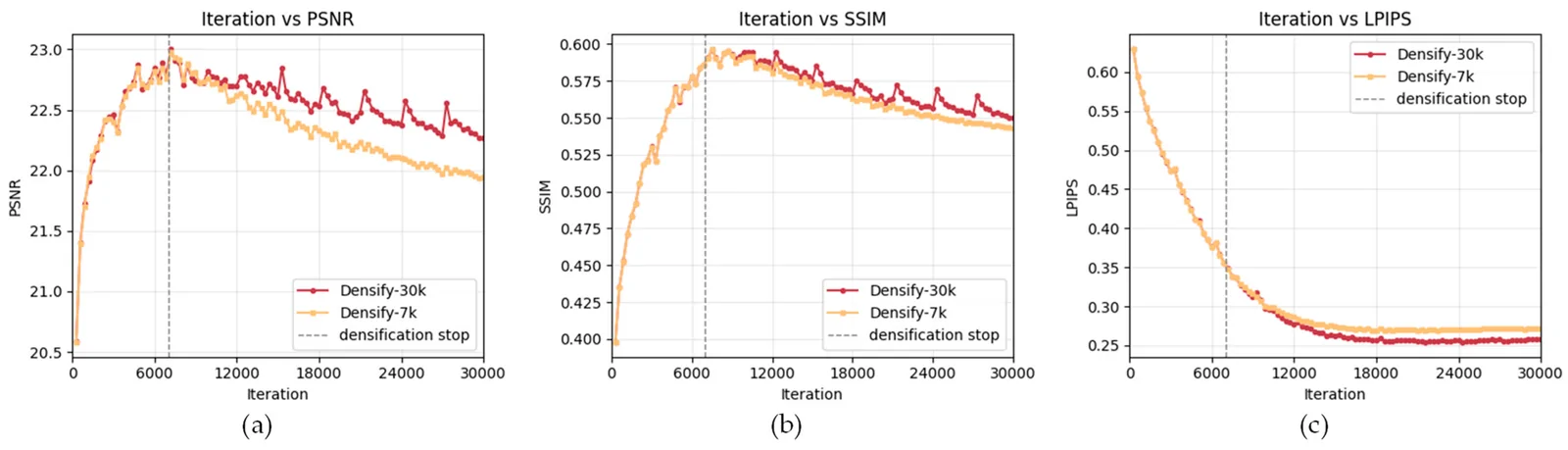
Comparison of rendering quality under the Densify-30k and Densify-7k conditions. (**a**) PSNR, (**b**) SSIM, and (**c**) LPIPS are shown as functions of training iteration. The dashed vertical line indicates iteration 7000, at which adaptive density control ended in the Densify-7k condition. PSNR and SSIM reached their peak values at approximately 7000–10,000 iterations and then gradually decreased under both conditions. After iteration 7000, LPIPS changed little under Densify-7k but continued to decrease slightly under Densify-30k. Overall, the differences between the two conditions remained small.

Figure 4 shows the changes in the quality metrics for the test images under the Densify-7k and Densify-30k conditions. Under both conditions, PSNR and SSIM increased rapidly during the early stage of training, reached values close to their maxima at approximately 7000 iterations, and then gradually decreased. At iteration 30,000, the three-run mean ± standard deviation values of PSNR, SSIM, and LPIPS were 22.2030 ± 0.0547 dB, 0.5482 ± 0.0007, and 0.2862 ± 0.0007, respectively, for Densify-30k, and 21.9639 ± 0.0404 dB, 0.5422 ± 0.0007, and 0.3012 ± 0.0002 for Densify-7k. Thus, extending adaptive density control improved the mean PSNR by 0.2391 dB and SSIM by 0.0060, while reducing LPIPS by 0.0150. The small standard deviations indicate limited run-to-run variability, and the improvements remained modest across all three test-view metrics.

**Figure 5:**
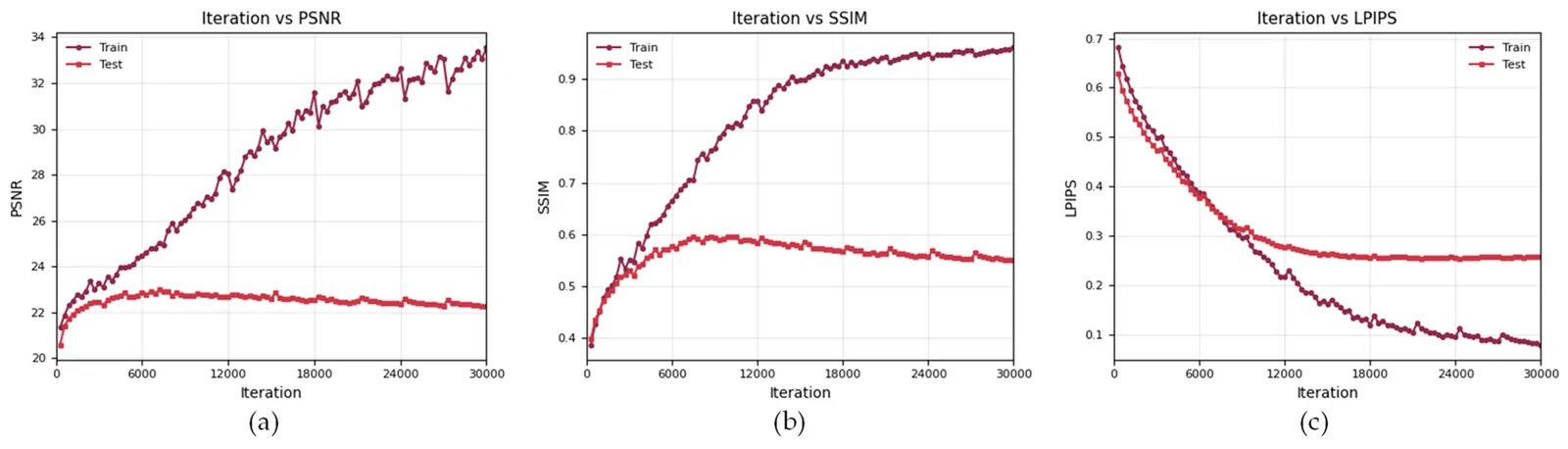
Comparison of training- and test-view rendering quality during optimization under the Densify-30k condition. (**a**) PSNR, (**b**) SSIM, and (**c**) LPIPS are shown as functions of training iteration. The training-view metrics continue to improve throughout optimization, whereas the test-view PSNR and SSIM approach saturation in the middle stage and then slightly decrease. Test-view LPIPS improves mainly during the early to middle stages and changes only slightly thereafter. These trends indicate that continued optimization improves fitting to the training views but does not necessarily lead to further improvement in held-out test-view quality.

Figure 5 replots the Densify-30k test-view metrics shown in Figure 4 together with the corresponding training-view metrics. For the training images, PSNR and SSIM continued to increase until iteration 30,000, whereas LPIPS continued to decrease. In contrast, the test-view metrics plateaued after the middle stage of training, and PSNR and SSIM gradually decreased. This divergence suggests that later-stage optimization improved the fit to the training views but contributed little to held-out test-view quality.

Combined with the results of the previous section, the Densify-30k condition produced approximately 1.9 times as many Gaussians and required approximately 1.5 times the cumulative processing time of the Densify-7k condition, whereas the final differences in test-image quality were small. Under the present experimental conditions, the additional quality improvement obtained through continued densification after the middle stage of training was therefore limited relative to the increase in computational cost.

### 4.3. Spatial Distribution and Convergence of Image Errors

**Figure 6:**
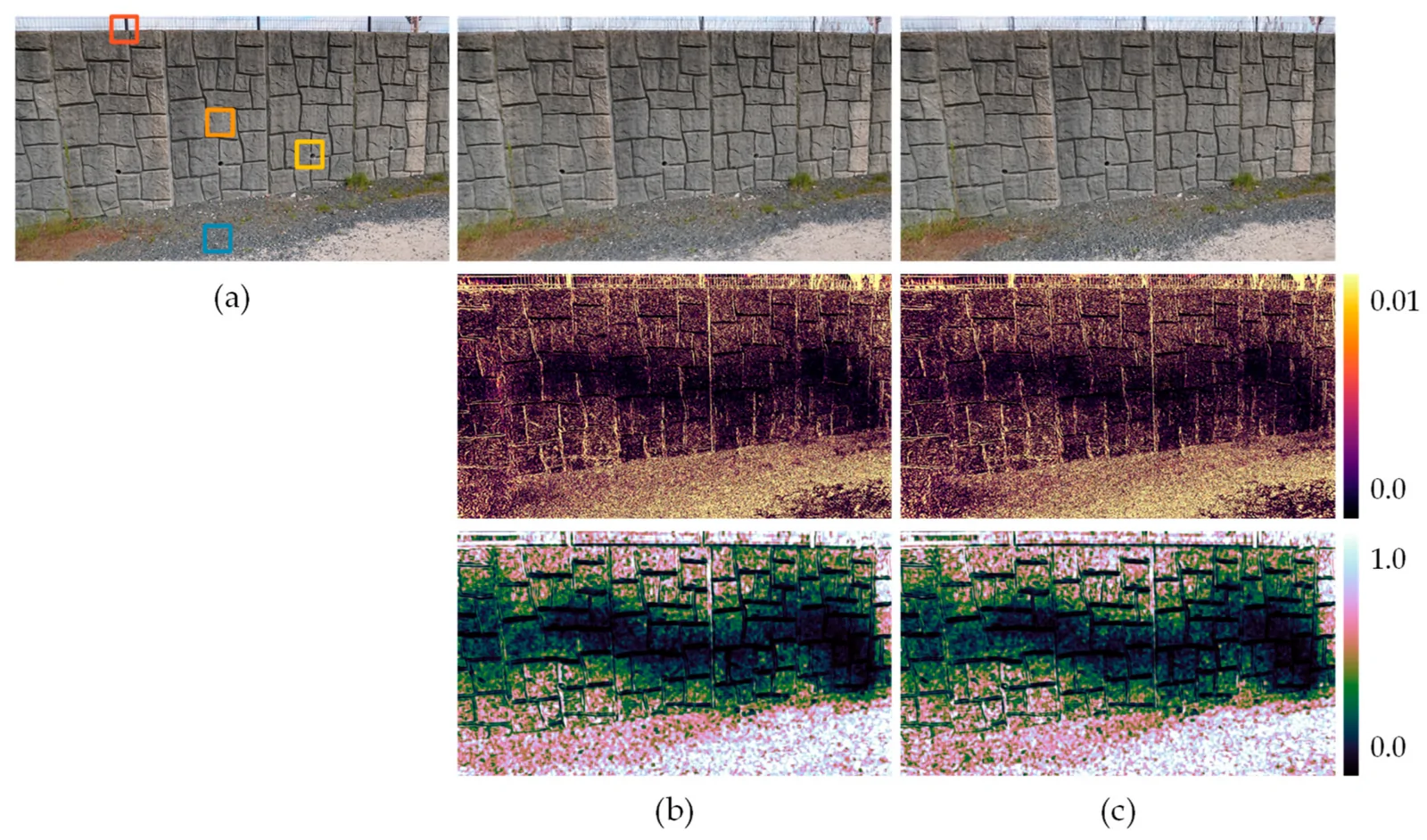
Visual comparison of reconstruction results and image error maps for the wall scene. (**a**) Reference image, where the colored rectangles indicate the regions used for the magnified comparisons. (**b**) Densify-30k condition and (**c**) Densify-7k condition at iteration 30,000. For (**b**,**c**), the top row shows the rendered image, the middle row shows the pixel-wise RGB MSE map, and the bottom row shows the local SSIM error map. Both conditions reproduce the overall wall texture and block structure similarly. Relatively high-error regions are mainly observed in the gravel and vegetation areas, in the background, around the fence, and locally around fine wall details such as block boundaries. The spatial distribution of errors is similar between the two conditions, indicating that extending adaptive density control beyond iteration 7000 does not substantially change the local error pattern in this scene.

Figure 6 shows the reference image, the rendered images, and the corresponding error maps at iteration 30,000 for the Densify-30k and Densify-7k conditions. Under both conditions, the interior of the wall was generally reproduced well. Regions with high pixel-wise RGB MSE and local SSIM error were mainly distributed over soil, gravel, and grass surfaces, the background, areas around the fence, and boundary regions such as the upper edge of the wall and the joints between the stone blocks. However, no substantial difference was observed between the two conditions in either the locations of high-error regions or the overall spatial distribution of errors. Thus, although extending adaptive density control beyond iteration 7000 produced a slight improvement in image quality for this scene, it did not substantially change the regions in which errors remained.

**Figure 7:**
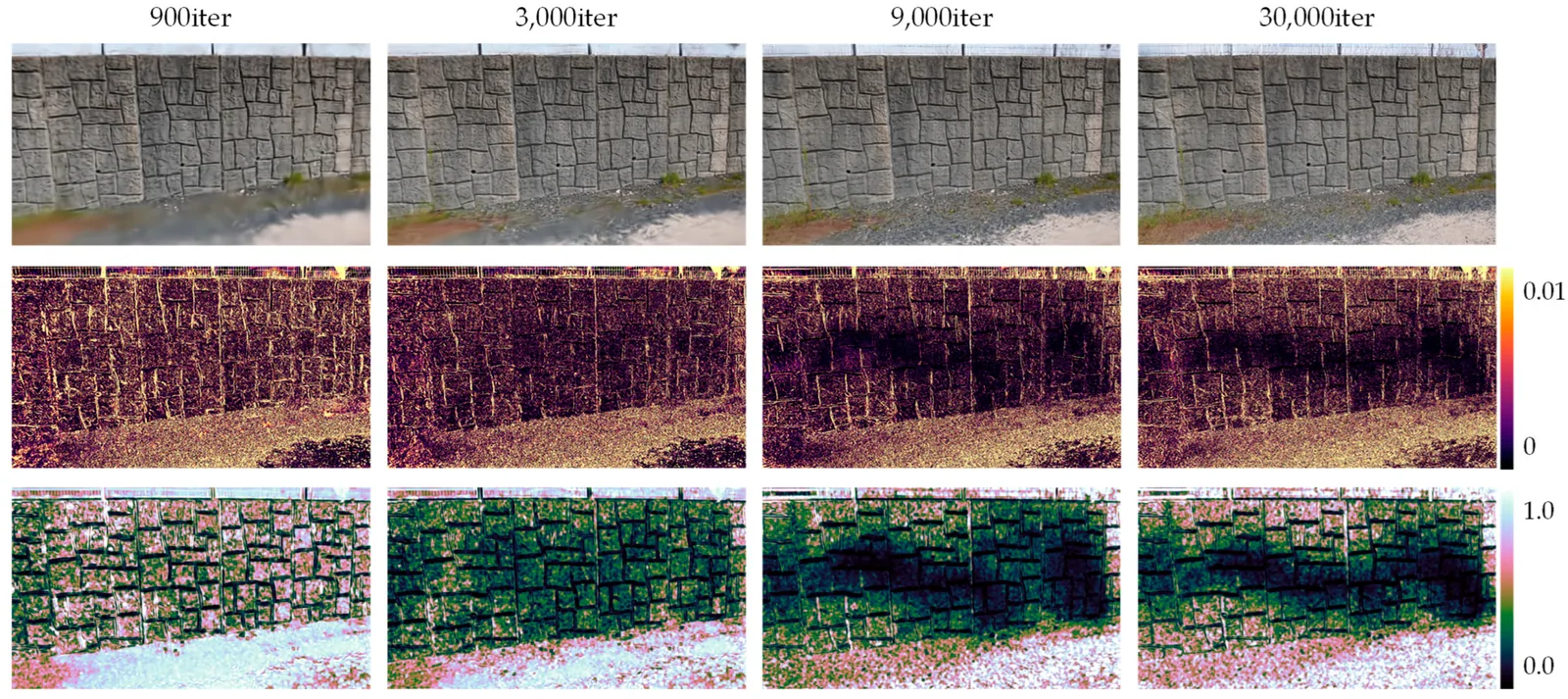
Spatial error distributions during optimization under the Densify-30k condition. The columns correspond to 900, 3000, 9000, and 30,000 iterations, and the rows show the rendered images, pixel-wise RGB MSE maps, and local SSIM error maps, respectively. The color scales range from 0 to 0.01 for MSE and from 0 to 1.0 for local SSIM error.

Figure 7 shows the rendered images and corresponding error maps at iterations 900, 3000, 9000, and 30,000 under the Densify-30k condition. At iteration 900, substantial blurring was observed over the wall and ground surfaces. Rendering quality improved markedly between iterations 3000 and 9000, accompanied by reductions in both MSE and local SSIM error, whereas changes after iteration 9000 were limited. Residual errors remained mainly in nonuniform ground textures, around the background and fence, and in fine wall details such as joints and small holes.

**Figure 8:**
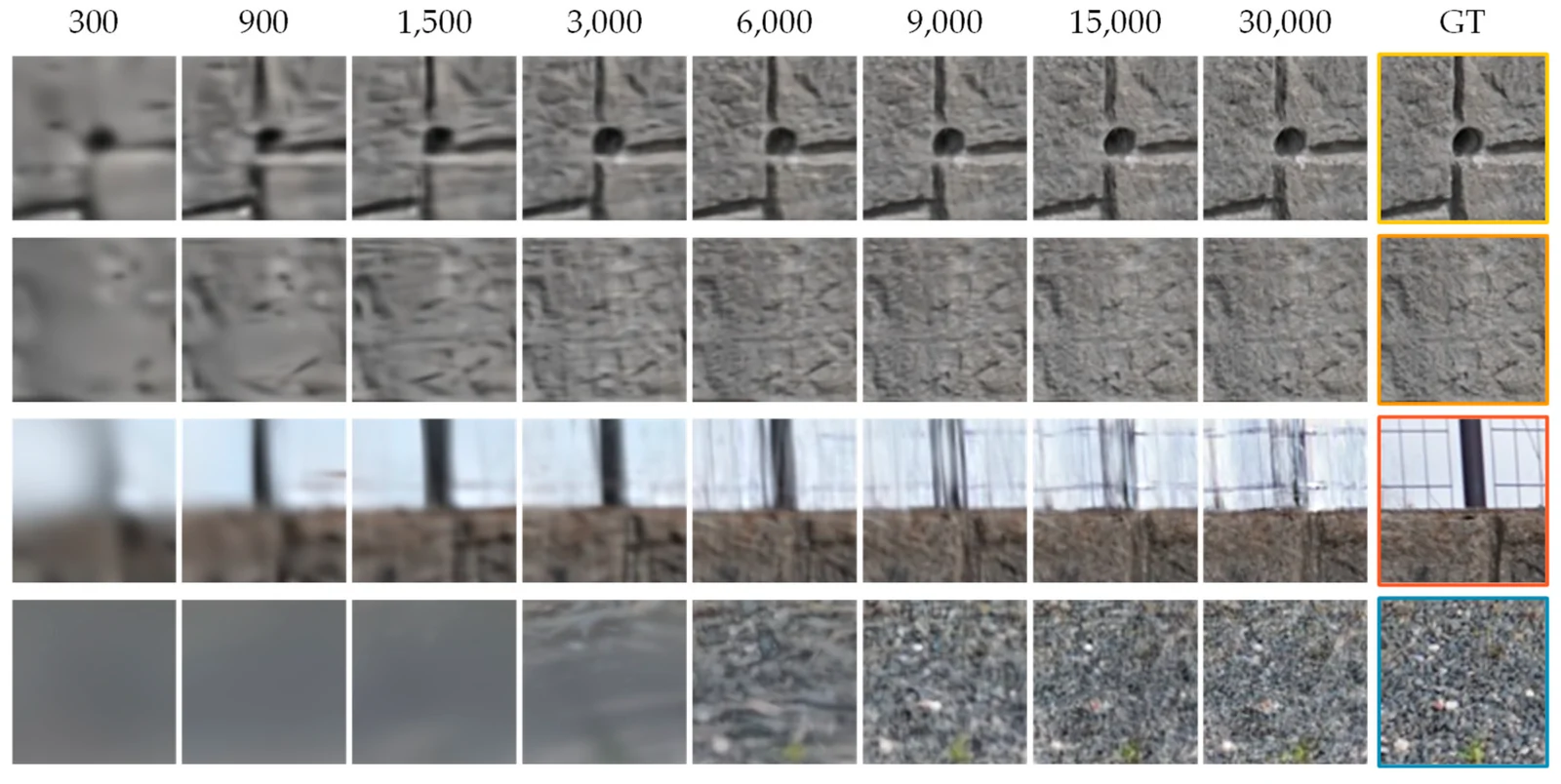
Local rendering results for representative wall-scene regions at different training iterations under the Densify-30k condition. The columns correspond to 300, 900, 1500, 3000, 6000, 9000, 15,000, and 30,000 iterations, followed by the ground-truth image (GT). The rows show a small hole, fine stone-surface texture, the upper wall boundary, and gravel texture, respectively.

Figure 8 presents magnified views of a small hole, the stone surface, the upper edge of the wall, and the gravel area. The outline of the small hole and the fine texture of the stone surface became clearer mainly between iterations 3000 and 9000. In contrast, blurring and color bleeding remained near the upper wall boundary and around the fence even at iteration 30,000. In the gravel area, the granular texture continued to improve during later training, but fine details remained less stable than those on the wall surface. These results indicate that local artifacts were more likely to persist around the upper wall boundary, the fence, and the gravel area than in the wall interior, which was consistently observed from multiple viewpoints and exhibited a relatively stable appearance.

Overall, most improvements in the spatial error distributions occurred by approximately 9000 iterations, while subsequent changes were limited. Extending adaptive density control beyond this stage did not substantially alter the regions in which local errors persisted, particularly around the upper wall boundary, the fence, and the gravel area.

### 4.4. Continuous Spatial Hold-Out Evaluation

To examine rendering quality under a more conservative train–test separation, nine consecutive wall-scene images near the middle of the acquisition sequence were excluded from 3DGS optimization and evaluated as a continuous spatial hold-out segment. Under the regular every-eighth split, the nearest training-camera distance for the nine test views had a mean of 0.370 m and a median of 0.406 m, whereas the continuous hold-out split increased these values to 1.304 m and 1.366 m, respectively. The maximum nearest-camera distance increased from 0.468 m to 2.325 m. The mean viewing-direction difference relative to the nearest-position training camera also increased from 2.00° to 3.13°, with corresponding median values of 1.68° and 2.82°.

The continuous hold-out condition yielded a PSNR of 16.5993 dB, an SSIM of 0.3438, and an LPIPS of 0.4264. For comparison, the regular every-eighth Densify-30k evaluation yielded 22.2030 ± 0.0547 dB, 0.5482 ± 0.0007, and 0.2862 ± 0.0007, respectively, over three runs. Thus, rendering quality decreased substantially when a continuous portion of the camera trajectory was excluded from training. Although the continuous hold-out experiment was conducted as a single run, the magnitude of this degradation was much larger than the run-to-run variability observed under the regular split.

The per-view results also showed a clear positional tendency within the held-out segment. PSNR was 19.54 and 19.79 dB near the two ends of the excluded segment, whereas it decreased to 13.93 dB near the center, where the nearest training-camera distance was greatest. Similar tendencies were observed for SSIM and LPIPS. These results indicate that the regular interval-based split provides a relatively favorable near-view interpolation condition for this wall sequence, whereas the continuous hold-out evaluation reveals substantially lower rendering quality when test viewpoints are more spatially separated from the training cameras.

## 5. Wall-Scene Case Study II: Gaussian Attribute Analysis

This section presents the results of the Gaussian attribute and local-error analyses described in Section 3.4 for the wall scene.

### 5.1. Evolution of Gaussian Scale Distribution

**Figure 9:**
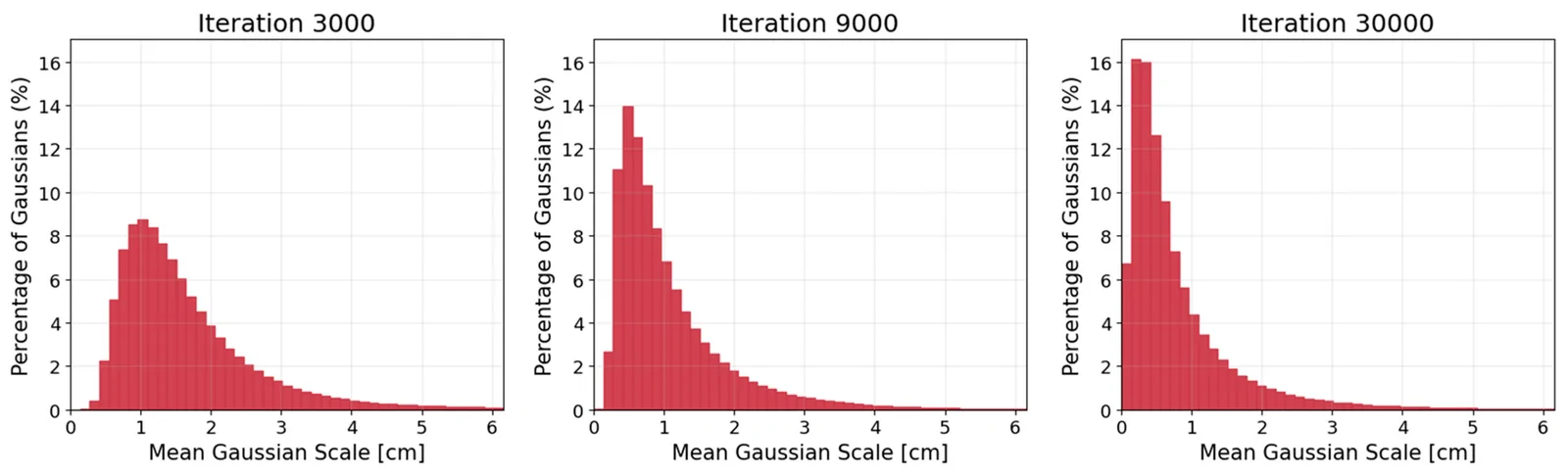
Evolution of Gaussian mean-scale distributions in approximate real-world units at selected training iterations. The panels correspond to iterations 3000, 9000, and 30,000. The horizontal axis indicates the mean Gaussian scale in centimeters, and the vertical axis indicates the percentage of Gaussians.

Figure 9 and Table 2 show the evolution of the Gaussian scale distribution and its summary statistics during training.

From iteration 1500 to iteration 30,000, the number of Gaussians increased from 445,114 to 8,328,905. In contrast, the mean scale decreased from 2.36 cm to 0.82 cm, the median from 1.85 cm to 0.53 cm, and the 95th percentile from 5.60 cm to 2.45 cm. Figure 9 also shows that the peak of the distribution shifted toward smaller scales and that the proportion of small Gaussians increased as training progressed.

The number of Gaussians reached 9,023,763 at iteration 15,000 and then decreased to 8,328,905 by iteration 30,000, indicating that, on balance, pruning removed more Gaussians than densification added during the later stage of training. Meanwhile, the mean, median, and 95th-percentile scales continued to decrease, indicating a continued shift toward smaller Gaussian scales. However, the 99th-percentile scale remained 4.45 cm at iteration 30,000, showing that relatively large Gaussians were still present in the upper tail of the distribution. The 99th percentile also increased slightly from 4.34 cm at iteration 15,000, indicating that the upper-tail scales did not decrease uniformly during the later stage of training.

### 5.2. Spatial Distribution of Scale–Opacity Classes

**Figure 10:**
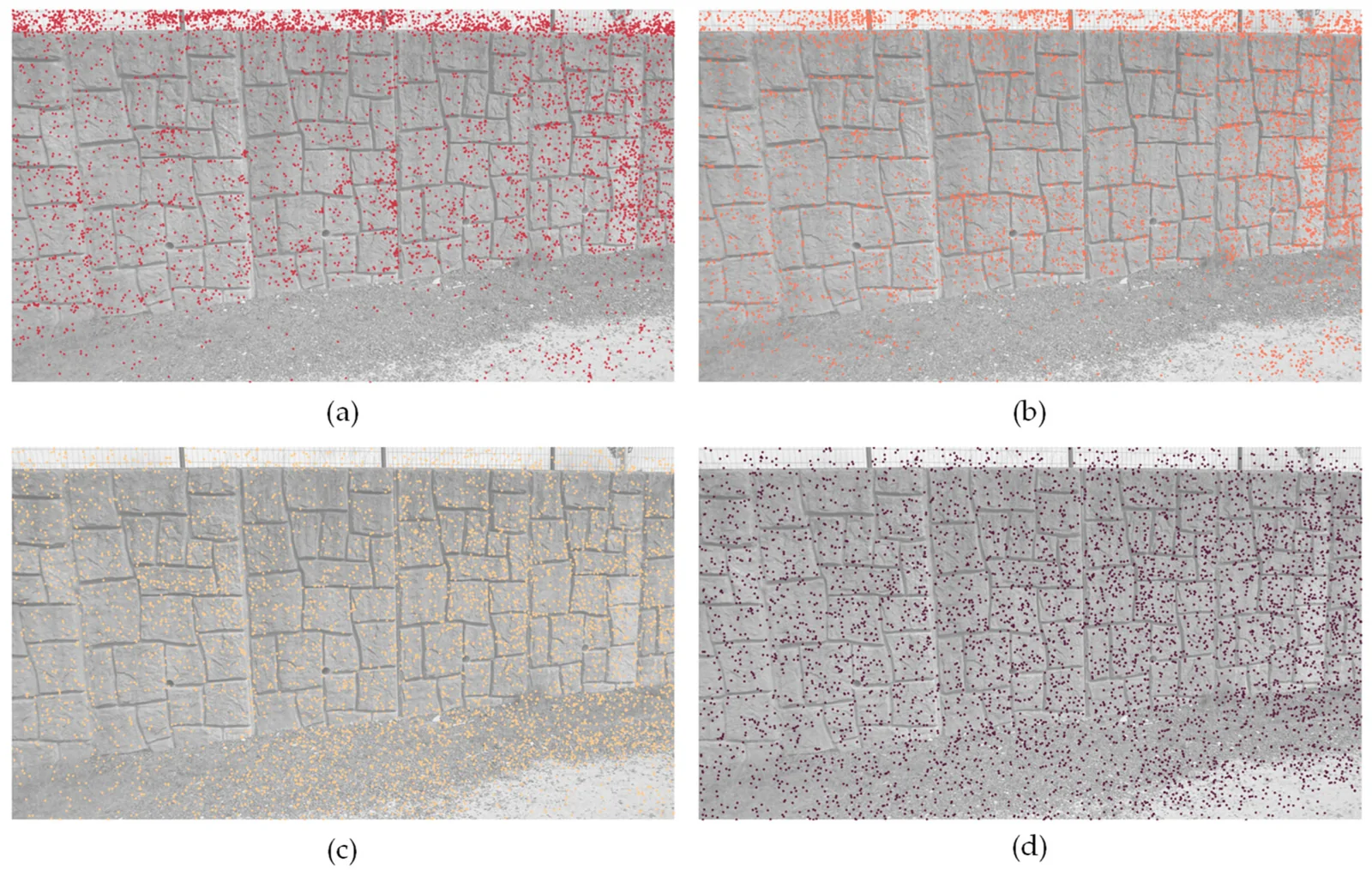
Image-space distributions of Gaussian centers classified by scale and opacity: (**a**) large + low opacity, (**b**) large + high opacity, (**c**) normal + low opacity, and (**d**) normal + high opacity. Large Gaussians were defined as the upper 5% of the mean-scale distribution, and the opacity classes were separated at the median learned opacity. Up to 5000 Gaussians were randomly sampled from each class and projected onto the representative test image. Only centers projected inside the image frame are shown; visibility and rendering contribution are not considered. The different colors are used only to visually distinguish the four scale–opacity classes.

Figure 10 shows the projected distributions of the four groups. Large-scale Gaussians, comprising the large + low-opacity and large + high-opacity groups, were projected less frequently onto the central wall region than normal-scale Gaussians, regardless of opacity. Instead, they were more frequently projected onto the fence near the upper edge of the image and onto background regions containing the sky and distant objects. Some were also projected onto soil, gravel, and grass areas, although less frequently than normal-scale Gaussians.

In contrast, normal-scale Gaussians, comprising the normal + low-opacity and normal + high-opacity groups, were distributed broadly across the image, including the central wall region and the soil, gravel, and grass areas.

Differences in projected locations attributable to opacity were limited. No clear spatial separation was observed between the normal + low-opacity and normal + high-opacity groups or between the large + low-opacity and large + high-opacity groups. Overall, spatial differences were more evident between the scale classes than between the opacity classes.

### 5.3. Relationship with Local MSE and SSIM Error

**Figure 11:**
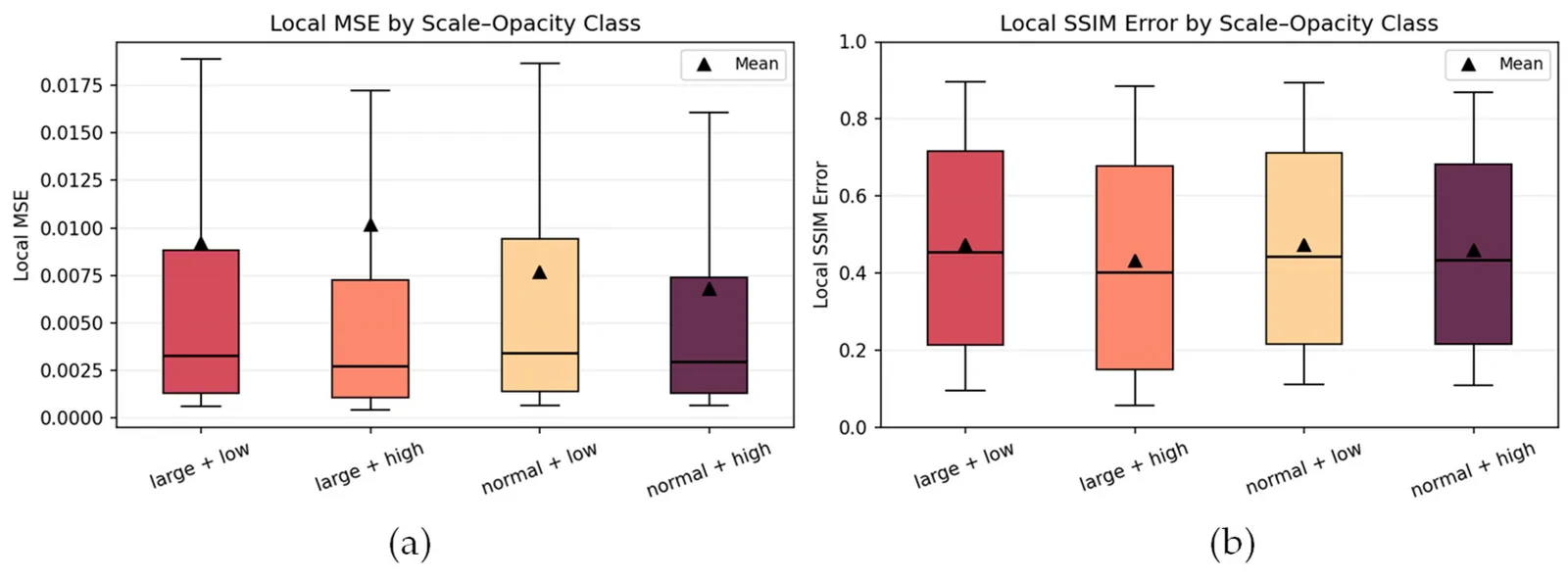
Distributions of local image errors for the four scale–opacity classes. (**a**) Local MSE and (**b**) local SSIM error evaluated around the projected Gaussian centers. The distributions substantially overlapped, and no clear separation was observed among the four classes. The boxes indicate Q1–Q3, the center lines indicate the medians, the whiskers indicate P10–P90, and the triangular markers indicate the means.

Figure 11 shows the distributions of local MSE and local SSIM error for the four scale–opacity groups. For both metrics, the distributions substantially overlapped, and no clear separation was observed among the groups. Thus, although the scale classes exhibited different spatial distributions in Figure 10, these differences did not translate into distinct patterns of local image-space error. The scale–opacity classification alone was therefore insufficient to identify high-error regions or artifacts. A sensitivity analysis using 3 × 3, 5 × 5, and 7 × 7 local-error aggregation windows produced the same qualitative result. For both local MSE and local SSIM error, the distributions of the four scale–opacity classes continued to overlap substantially, indicating that the lack of clear class separation was not specific to the 5 × 5 window.

The P95 scale threshold was adopted as an exploratory criterion rather than as a definitive artifact-detection threshold. Gaussian scale and learned opacity should therefore be treated as auxiliary descriptors and interpreted together with image-space error maps and ROI-level visual inspection.

## 6. Wall-Scene Case Study III: MVS-Relative Geometric Consistency

This section evaluates the relative geometric consistency of Gaussian centers with respect to the wall surface reconstructed by MVS. Because Gaussians are rendering primitives optimized for image reconstruction rather than surface samples, high rendering quality does not necessarily imply close spatial agreement with reference geometry. The MVS point cloud was generated from the same image sequence as the 3DGS model and is therefore not an independent ground truth; the results are interpreted as relative consistency rather than absolute surveying accuracy.

### 6.1. MVS Reference Point Cloud and Analysis Scope

**Figure 12:**
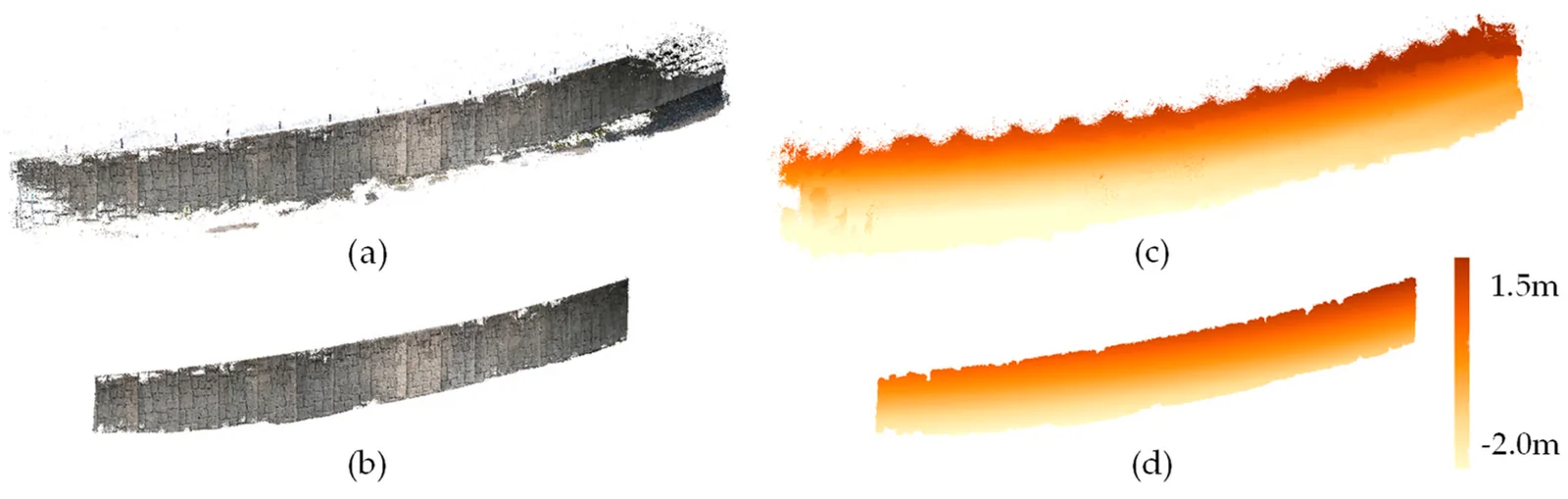
MVS point clouds and Gaussian-center distributions for the wall scene. (**a**) Original COLMAP MVS point cloud, including the wall, ground, fence, and surrounding regions. (**b**) Wall-reference point cloud obtained by extracting the wall region and resampling it at an approximately 1 cm spacing. (**c**) Gaussian centers of the final 3DGS model. (**d**) Wall-near Gaussians, defined as those with center-to-reference distances of 20 cm or less. The MVS point clouds in (**a**,**b**) are shown in reconstructed RGB colors, whereas the Gaussian centers in (**c**,**d**) are colored by their *y* coordinate using the same color scale. The colors in (**c**,**d**) are used only for visualization.

Figure 12 shows (a) the original MVS point cloud generated using COLMAP, (b) the extracted and resampled wall-reference point cloud, (c) the Gaussian centers of the final 3DGS model, and (d) the wall-near Gaussians used in the Gaussian–MVS distance analysis. The original MVS point cloud contained 9,051,756 points and included the wall, ground, fence, and surrounding areas. After manually extracting the wall region and resampling it at an approximately 1 cm spacing, the wall-reference point cloud contained 482,499 points.

The final 3DGS model at iteration 30,000 contained 8,328,905 Gaussians. Of these, 4,051,146 had centers located within 20 cm of the wall-reference point cloud and were defined as wall-near Gaussians. For each wall-near Gaussian, the Euclidean distance from its center to the nearest reference point was defined as the Gaussian–MVS distance. The uniform scale factor described in Section 3.3 was applied to the MVS points, Gaussian-center coordinates, and Gaussian scales.

### 6.2. Gaussian Scale and MVS-Relative Distance

**Figure 13:**
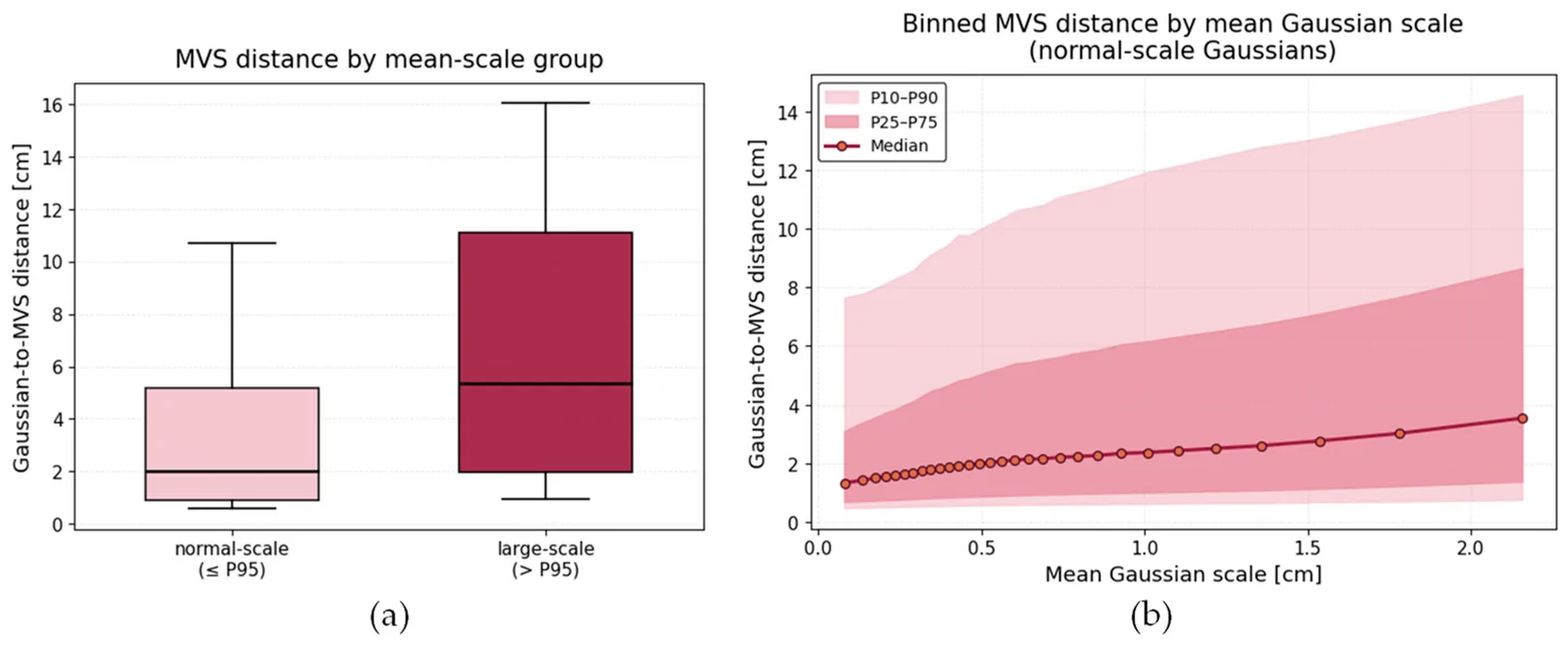
Relationship between Gaussian scale and Gaussian–MVS distance for wall-near Gaussians. (**a**) Distance distributions for normal- and large-scale Gaussians separated at the P95 scale threshold. (**b**) Median distance with P25–P75 and P10–P90 ranges for equal-count bins of normal-scale Gaussians. Larger scales were associated with greater Gaussian–MVS distances, although the distributions substantially overlapped.

Figure 13 shows the relationship between Gaussian scale and Gaussian–MVS distance for the wall-near Gaussians defined in Section 6.1. The 95th percentile of Gaussian scale was used as the threshold, with Gaussians at or below P95 classified as normal-scale Gaussians and those above P95 classified as large-scale Gaussians.

As shown in Figure 13a, large-scale Gaussians exhibited greater median Gaussian–MVS distances and a broader upper-tail distribution than normal-scale Gaussians. This indicates that, among the Gaussians located within 20 cm of the wall reference point cloud, large-scale Gaussians tended to have greater center-to-reference distances than normal-scale Gaussians.

Figure 13b shows the results obtained by dividing the normal-scale Gaussians into scale intervals. The median Gaussian–MVS distance increased gradually with Gaussian scale. However, the distance distributions within each interval showed substantial variation, and some normal-scale Gaussians were also located relatively far from the MVS wall surface.

Overall, Gaussian scale showed a limited association with Gaussian–MVS distance, but the distributions substantially overlapped. Because the distance was calculated from Gaussian centers and did not account for scale or anisotropy, a large value does not necessarily indicate an incorrect scene representation. A sensitivity analysis using P90, P95, and P99 scale thresholds showed the same qualitative tendency. The median Gaussian–MVS distance was consistently greater for the large-scale class than for the normal-scale class under all three thresholds, indicating that the observed association was not specific to the P95 criterion. This tendency was also maintained when the wall-near distance cutoff was varied to 10, 20, and 30 cm, with the large-scale class consistently showing greater median Gaussian–MVS distances than the normal-scale class. Thus, the observed scale–distance association was not specific to either the P95 scale threshold or the 20 cm wall-near cutoff.

### 6.3. MVS-Relative Distance by Scale–Opacity Class

**Figure 14:**
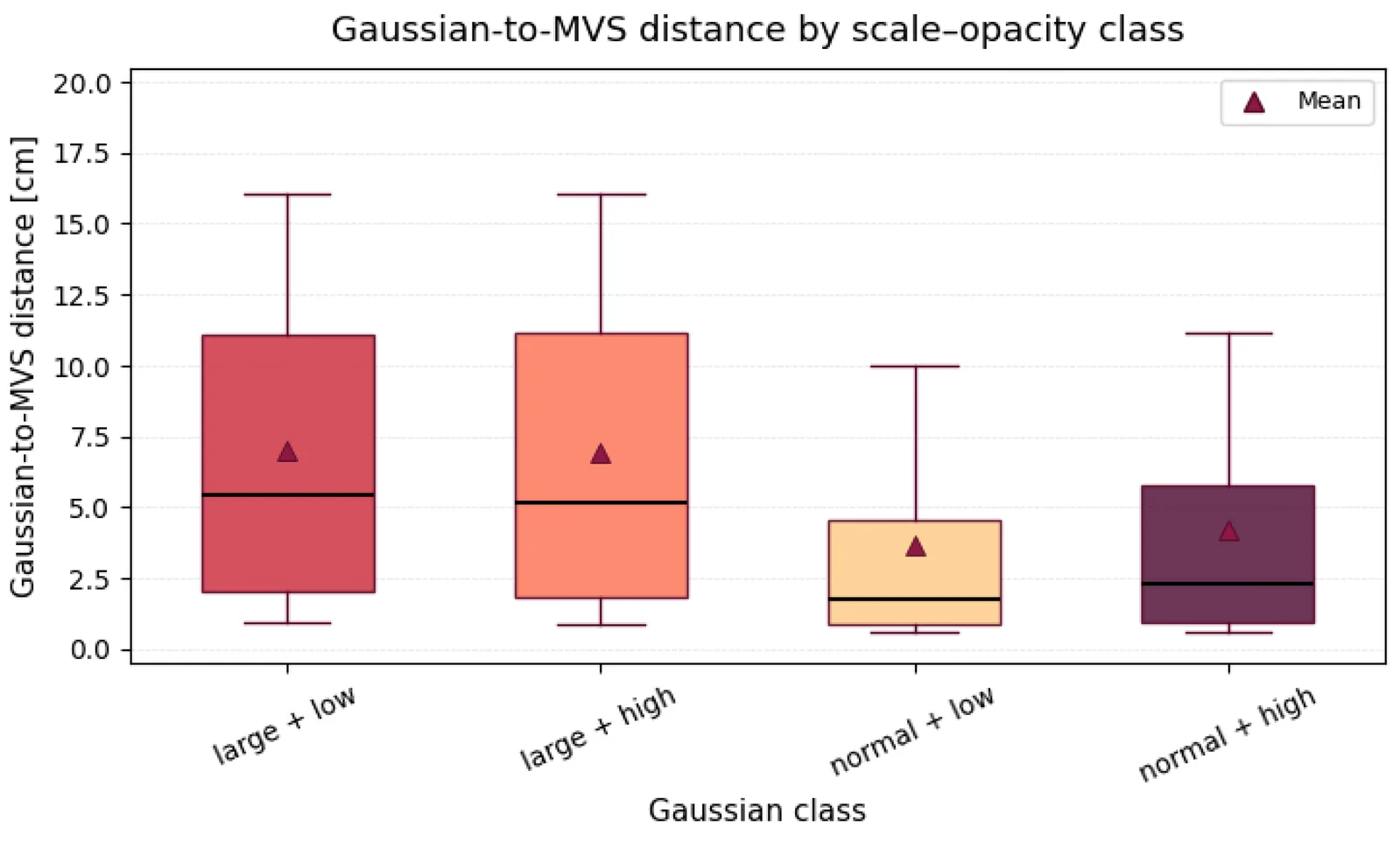
Gaussian–MVS distance distributions for the four scale–opacity classes of wall-near Gaussians. Classes were defined using the P95 threshold of mean Gaussian scale and the median learned opacity of the final model. Large-scale classes showed greater Gaussian–MVS distances than normal-scale classes, whereas differences between low- and high-opacity classes were limited.

Figure 14 shows the Gaussian–MVS distance distributions for the four scale–opacity classes. Both large-scale classes exhibited greater median distances and broader upper-tail distributions than the normal-scale classes. In contrast, within each scale category, the differences between the low- and high-opacity classes were small relative to the differences between the scale classes. A sensitivity analysis using the 25th, 50th, and 75th percentiles of learned opacity as thresholds showed that the ordering between low- and high-opacity Gaussians depended on the scale class and threshold. In particular, the ordering reversed across opacity thresholds for the large-scale class, whereas high-opacity Gaussians showed only slightly greater median distances within the normal-scale class.

Thus, Gaussian–MVS distance was more clearly associated with Gaussian scale than with learned opacity, although the distributions substantially overlapped. In Section 5.3, neither local MSE nor local SSIM error showed clear separation among the four classes. These results indicate that Gaussian scale and learned opacity provide only auxiliary information: Gaussian scale was associated with MVS-relative distance, whereas neither attribute clearly distinguished local image-space errors. Neither attribute alone was sufficient to determine image-space error or MVS-relative spatial agreement.

### 6.4. Cross-Sectional Distribution of Gaussian Centers

The Gaussian–MVS distances used in Section 6.2 and Section 6.3 were unsigned and therefore did not indicate on which side of the wall the Gaussian centers were located. To examine their front-to-back distribution, a representative horizontal slice was analyzed using a fitted wall profile and signed distances from the Gaussian centers.

**Figure 15:**
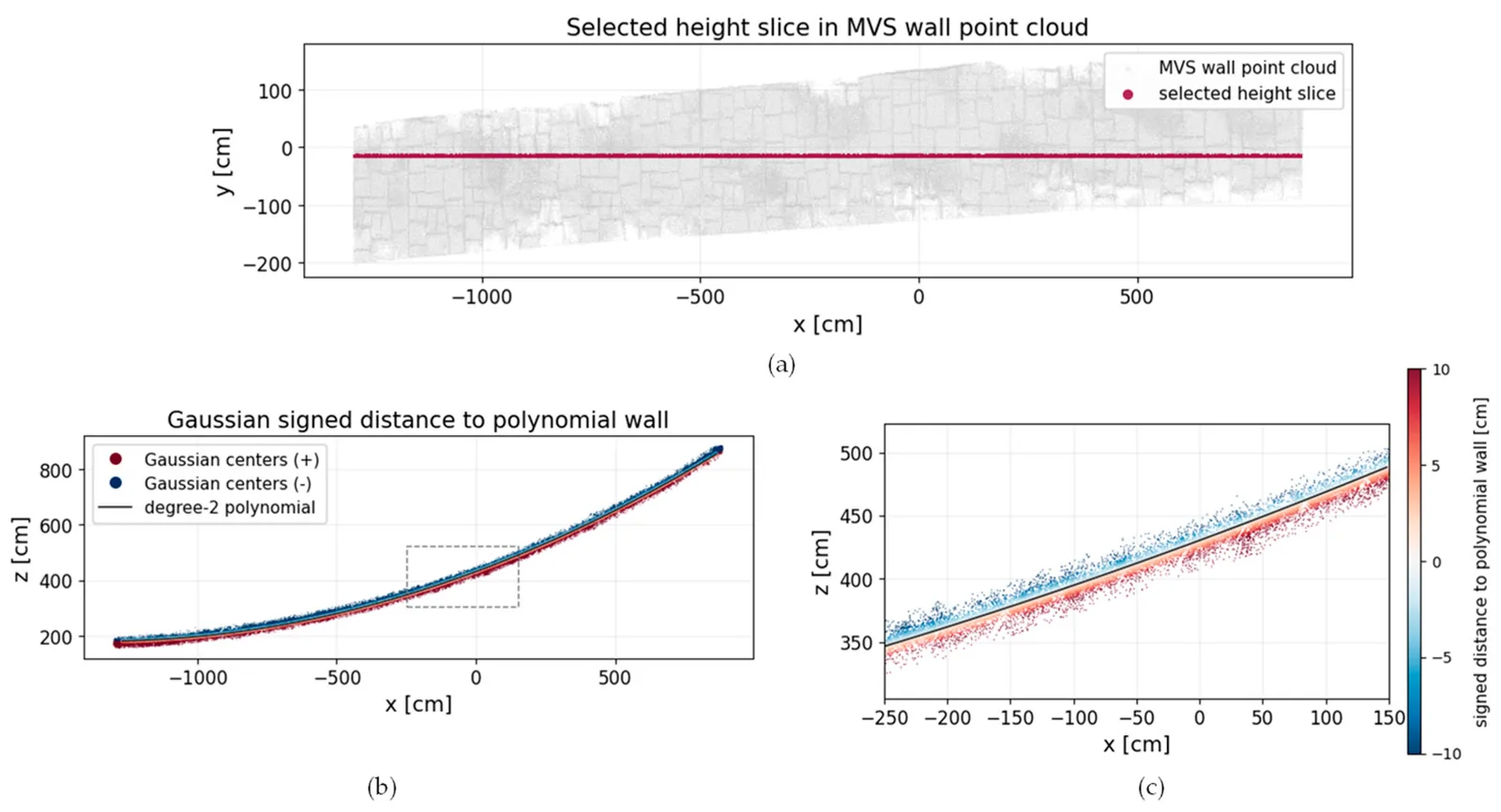
Cross-sectional distribution of wall-near Gaussian centers relative to a fitted quadratic wall profile. (**a**) MVS wall point cloud shown in gray, with points located within ±3 cm of the median *y*-coordinate highlighted in magenta. (**b**) Wall-near Gaussian centers within the same *y*-range, shown in the x–z plane and colored by their signed distance to the fitted profile; the front and rear sides of the wall were defined as positive and negative, respectively. (**c**) Enlarged view of the dashed region in (**b**). Panels (**b**,**c**) use the same color scale, limited to ±10 cm for visualization. The dashed rectangle in panel (**b**) indicates the region enlarged in panel (**c**), and the solid line represents the fitted quadratic wall profile.

Figure 15a shows a 6 cm thick slice centered at the median *y*-coordinate of the MVS wall point cloud in the x–y plane. The Gaussian centers within the same *y*-range were extracted from the wall-near subset defined by a three-dimensional Gaussian–MVS distance of 20 cm or less. The selected MVS points and Gaussian centers were then projected onto the x–z plane for the cross-sectional analysis. A quadratic polynomial was fitted to approximate the wall profile in this cross-section. To evaluate the goodness of fit, the absolute distances of the MVS points from the fitted curve were calculated. Their median and 95th percentile were approximately 1.86 and 5.30 cm, respectively.

For the selected wall-near Gaussian centers, signed distances from the fitted curve were calculated to examine their front-to-back distribution. Figure 15b shows the Gaussian centers colored according to these signed distances. The front side of the wall was defined as positive and the rear side as negative. The signed distances had a mean of 0.07 cm, a standard deviation of 6.36 cm, a median of 0.11 cm, and a 5th–95th percentile range of −9.86 to 10.22 cm. The mean and median values close to zero indicate that the Gaussian centers were not systematically biased toward either side of the fitted wall profile. At the same time, the width of the percentile range indicates that they formed a spatially distributed band around the MVS wall cross-section. A sensitivity analysis using slice widths of 4, 6, and 8 cm produced the same qualitative interpretation. Across all three widths, the signed-distance medians remained close to zero, and the distribution widths were similar, indicating that the absence of a systematic front-to-back bias was not specific to the 6 cm slice.

The enlarged view in Figure 15c shows that most Gaussian centers within the selected slice were concentrated near the fitted curve, although some were located more than 10 cm away. For these 103,048 wall-near Gaussian centers, the absolute distances to the fitted polynomial profile had a median of 2.80 cm, a 90th percentile of 10.04 cm, and a 95th percentile of 14.05 cm. For the same Gaussian set, the three-dimensional nearest-neighbor distances to the full MVS wall reference point cloud had a median of 1.39 cm, a 90th percentile of 7.17 cm, and a 95th percentile of 9.96 cm. The distances to the polynomial profile were somewhat larger because the fitted curve represented a smoothed approximation of the wall cross-section, whereas the nearest-neighbor distances were measured directly to the MVS reference points. Nevertheless, both measures indicated spatial variations of a similar order of magnitude.

### 6.5. Image-Space Error and MVS-Relative Distance

**Figure 16:**
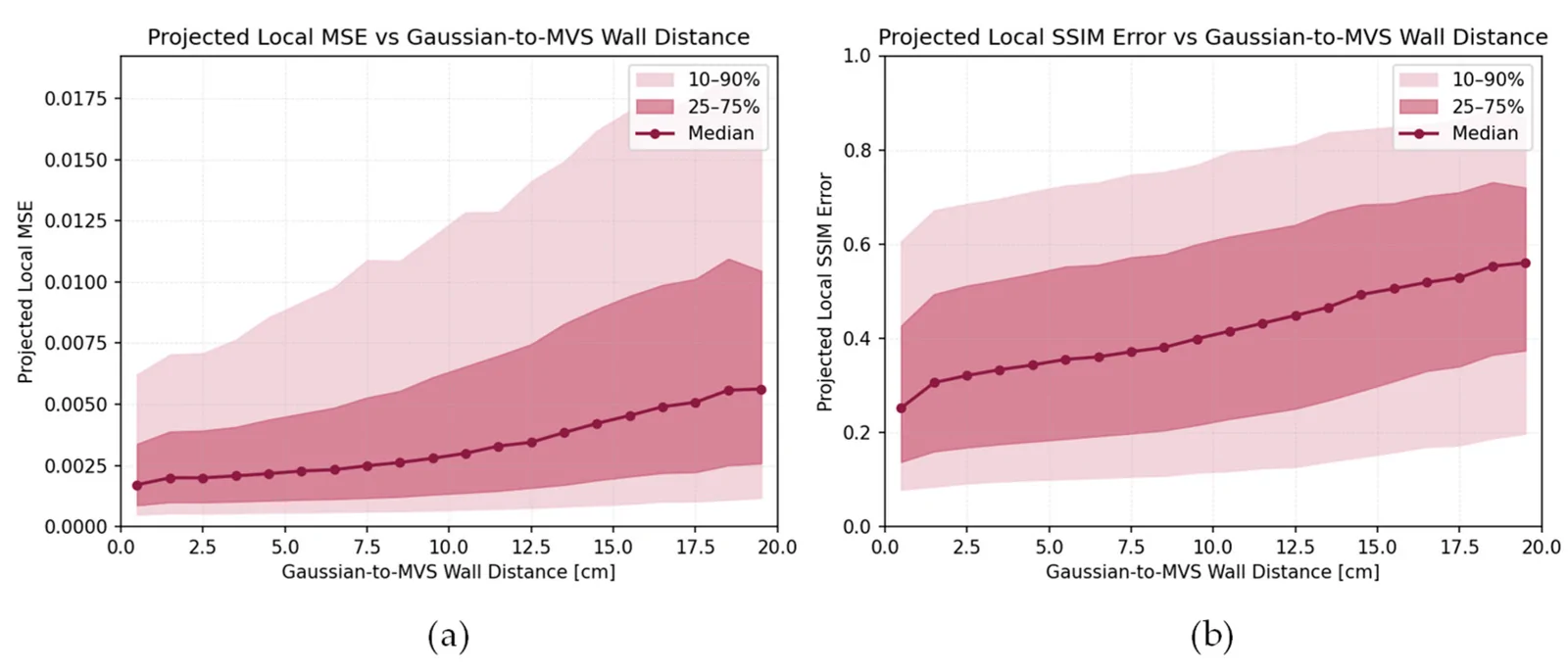
Relationship between Gaussian–MVS distance and projected local image error. (**a**) Projected local MSE and (**b**) projected local SSIM error aggregated by Gaussian–MVS distance bins. Lines with markers indicate median values, and shaded regions indicate the 25th–75th and 10th–90th percentile ranges. The median values of both metrics generally increased with Gaussian–MVS distance, although the percentile ranges substantially overlapped across the distance bins.

Figure 16a,b show the relationships of Gaussian–MVS distance with projected local MSE and projected local SSIM error, respectively. For both metrics, the median values generally increased with Gaussian–MVS distance. However, the percentile ranges remained broad and substantially overlapped across the distance bins.

These results indicate that greater Gaussian–MVS distance was associated with higher local image-space error in this wall scene. Nevertheless, the substantial overlap among the error distributions shows that Gaussian–MVS distance alone was insufficient to predict local image-space error reliably. Thus, image-space error and MVS-relative spatial consistency were related but remained distinct and complementary evaluation aspects.

## 7. Cross-Scene Assessment of Reconstruction Characteristics

This section presents a descriptive cross-scene assessment of the forest, piled-pier underside, steel-girder bridge, asphalt pavement, and outdoor sculpture scenes; the scene-specific acquisition conditions are summarized in Table 1. For the steel-girder bridge scene, the Bridge-200 condition, consisting of 200 training images and 9 test images, was used as the representative condition. The effects of reducing the training set to 100 and 50 images are examined separately in Section 7.7.

### 7.1. Forest Scene

**Figure 17:**
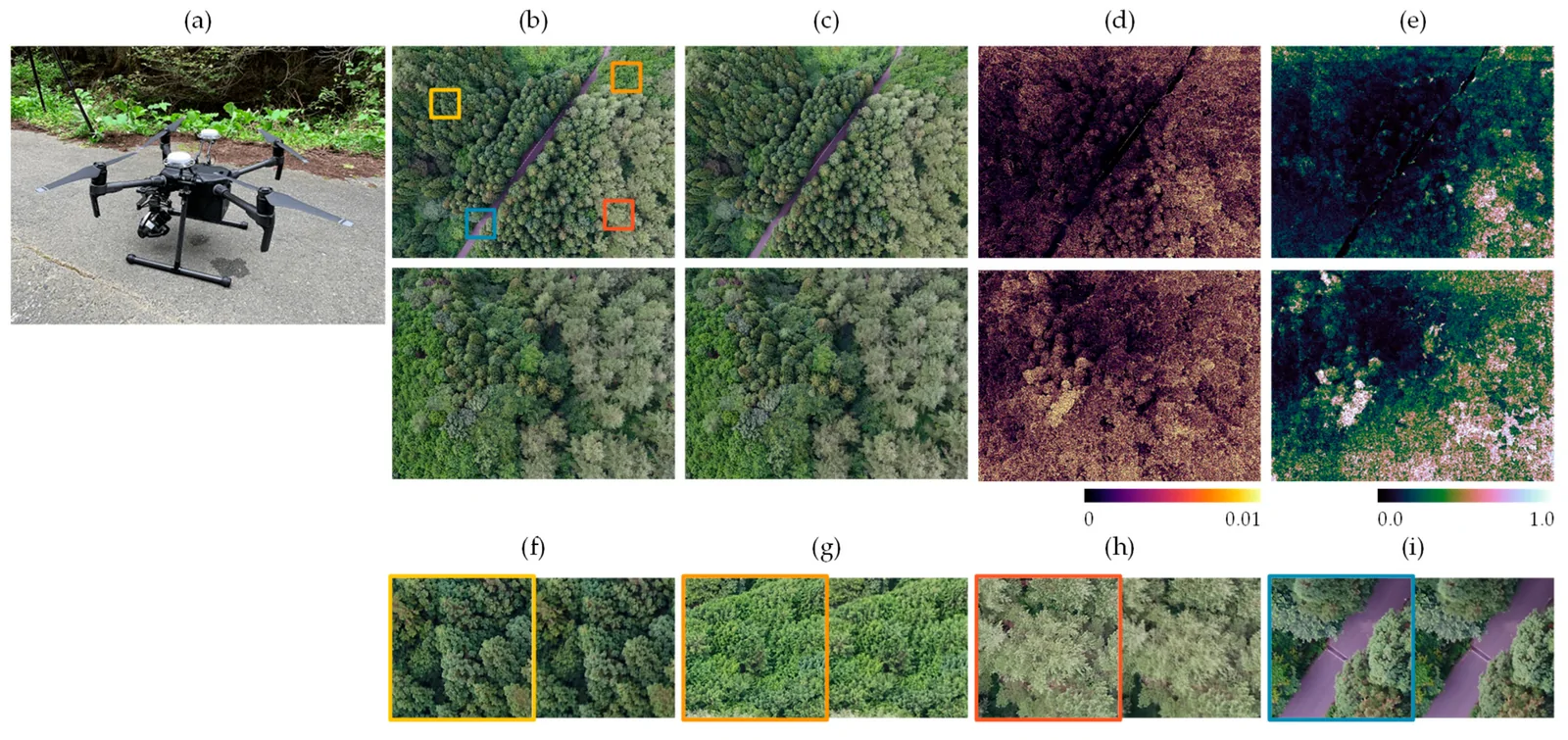
Forest-scene reconstruction results. (**a**) UAV-based image acquisition; (**b**) reference images with the selected ROIs; (**c**) 3DGS-rendered images; (**d**) pixel-wise RGB MSE maps; and (**e**) local SSIM error maps. The color scales range from 0 to 0.01 for MSE and from 0 to 1.0 for local SSIM error. Enlarged reference–rendering comparisons are shown for (**f**) a Japanese cedar (sugi) crown, (**g**) a hinoki cypress crown, (**h**) a Japanese red pine (akamatsu) crown, and (**i**) the road boundary. In each enlarged comparison, the reference image is shown on the left and the corresponding rendering on the right. The colored rectangles indicate the ROIs corresponding to the enlarged comparisons in panels (**f**–**i**).

The forest scene was reconstructed from nadir UAV imagery and included crowns of Japanese cedar, hinoki cypress, and Japanese red pine, as well as shadowed areas and boundaries between tree stands and roads. Because the images were acquired from an overhead viewpoint, mainly the upper crown surfaces were visible, whereas internal branches, trunks, and the ground were rarely observed beneath dense foliage. Figure 17 shows the reconstruction results for this scene.

The 3DGS rendering reproduced the overall crown shapes, tree distribution, dominant color patterns, and road geometry well. In the enlarged comparisons, the densely foliated Japanese cedar crown in panel (f) and hinoki cypress crown in panel (g) were reproduced relatively clearly, reflecting the consistent observation of their upper crown surfaces across the image sequence. In contrast, the Japanese red pine crown in panel (h) showed more noticeable blurring in the distribution of branches and foliage. Its lower foliage density made gaps between branches and leaves and dark interior regions more visible, and relatively large errors were also observed in both the pixel-wise RGB MSE and local SSIM error maps. The road boundary in panel (i) was generally preserved. These differences should not be attributed directly to tree species, but rather to foliage density, crown-surface continuity, visibility into the crown interior, and shadow conditions.

Overall, standard 3DGS represented crown-scale shapes and tree distributions well, whereas reconstruction was less stable in sparsely foliated crowns containing visible gaps and shadowed interior regions.

### 7.2. Pier Scene

**Figure 18:**
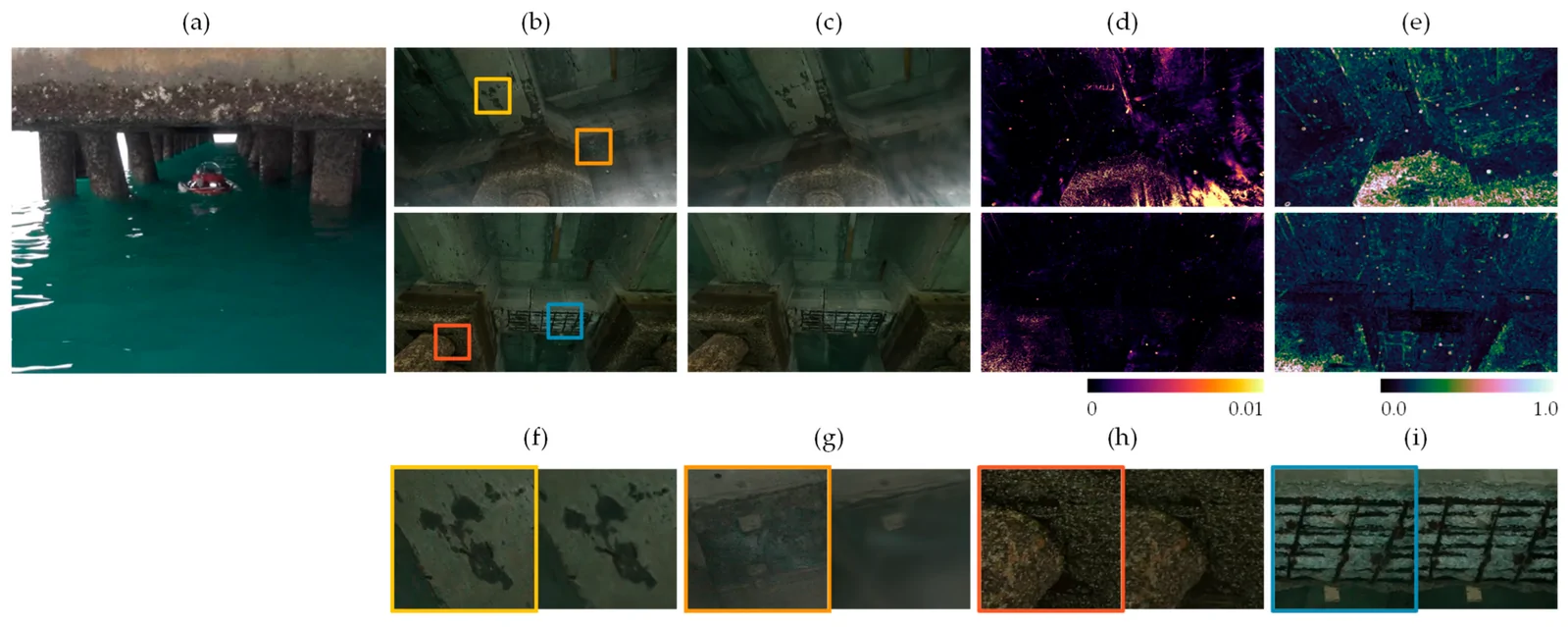
Piled-pier underside reconstruction results. (**a**) Boat-based image acquisition system; (**b**) reference images with the selected ROIs; (**c**) 3DGS-rendered images; (**d**) pixel-wise RGB MSE maps; and (**e**) local SSIM error maps. The color scales range from 0 to 0.01 for MSE and from 0 to 1.0 for local SSIM error. Enlarged reference–rendering comparisons are shown for (**f**) leakage stains, (**g**) surface discoloration, (**h**) rough concrete texture, and (**i**) exposed reinforcement. In each enlarged comparison, the reference image is shown on the left and the corresponding rendering on the right. The colored rectangles indicate the ROIs corresponding to the enlarged comparisons in panels (**f**–**i**).

The piled-pier underside scene included major structural components such as the concrete deck slab, beams, piles, and pile heads, together with water-leakage traces, surface discoloration, rough concrete textures, spalling-like defects, and exposed reinforcement. Images were acquired beneath the pier using a mobile imaging system mounted on a boat and equipped with LED lighting, a camera, and a gimbal [31,32]. Although the interior was generally dark, sunlight entered through the outer edges, producing large brightness variations within the images. Figure 18 shows the reconstruction results for this scene.

The 3DGS rendering generally reproduced the major structural components, including the underside of the deck slab, beams, pile heads, and side walls. In the enlarged comparisons, the leakage stain in panel (f) was slightly blurred, but its overall shape remained identifiable. The surface discoloration in panel (g) was more noticeably blurred. The rough concrete texture in panel (h) was reproduced relatively well without substantial degradation, and the exposed reinforcement in panel (i) remained clearly visible. These local features should therefore be verified using the corresponding ROIs in the original images and the error maps when 3DGS is used for inspection support.

### 7.3. Steel Girder Bridge Scene

**Figure 19:**
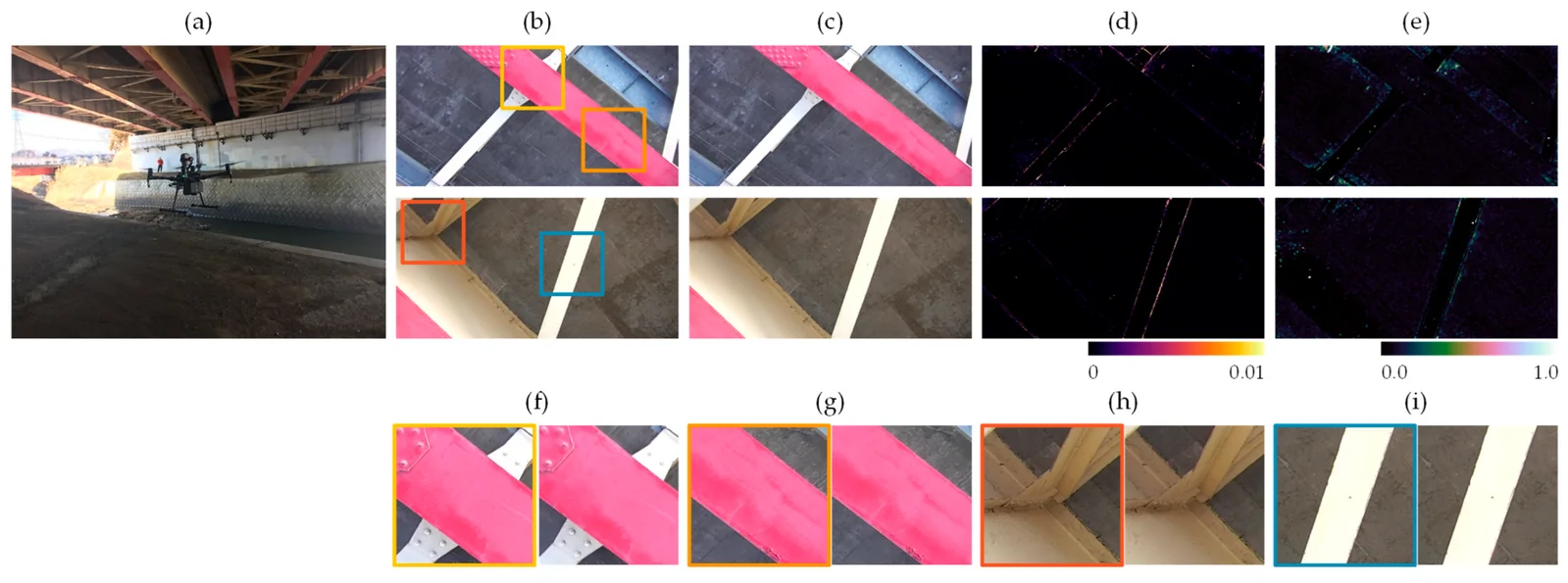
Steel-girder bridge reconstruction results. (**a**) UAV-based image acquisition; (**b**) reference images with the selected ROIs; (**c**) 3DGS-rendered images; (**d**) pixel-wise RGB MSE maps; and (**e**) local SSIM error maps. The color scales range from 0 to 0.01 for MSE and from 0 to 1.0 for local SSIM error. Enlarged reference–rendering comparisons are shown for (**f**) a boundary between red and white members with a fastener detail, (**g**) localized coating deterioration on the red girder, (**h**) a connection between overlapping steel members, and (**i**) small spot-like features on the deck underside. In each enlarged comparison, the reference image is shown on the left and the corresponding rendering on the right. The colored rectangles indicate the ROIs corresponding to the enlarged comparisons in panels (**f**–**i**).

The Bridge-200 condition was used as the representative setting for the steel-girder bridge scene. The scene included red- and white-painted steel members, splice plates, bolts and rivets, the gray underside of the deck slab, narrow gaps, repeated structural elements, shadowed regions, and localized coating deterioration. Multiple components with different colors, depths, and surface conditions overlapped within the same views, producing frequent occlusions and boundary transitions. Figure 19 shows the reconstruction results.

The 3DGS rendering generally reproduced the overall arrangement of the bridge underside, the major structural members, the dominant colors of the red and white members, and the shading of the deck underside. Slightly elevated local errors were observed mainly along the boundaries between structural members and around overlapping connection regions.

The magnified views showed that all four representative regions were reconstructed clearly under the Bridge-200 condition. The fastener detail and member boundary in panel (f), the localized coating deterioration in panel (g), the overlapping steel connection in panel (h), and the small spot-like feature on the deck underside in panel (i) remained identifiable and were reproduced at locations consistent with the reference images. These results indicate that, under the Bridge-200 condition, standard 3DGS preserved both major structural relationships and the selected local features examined in this scene.

### 7.4. Asphalt Scene

**Figure 20:**
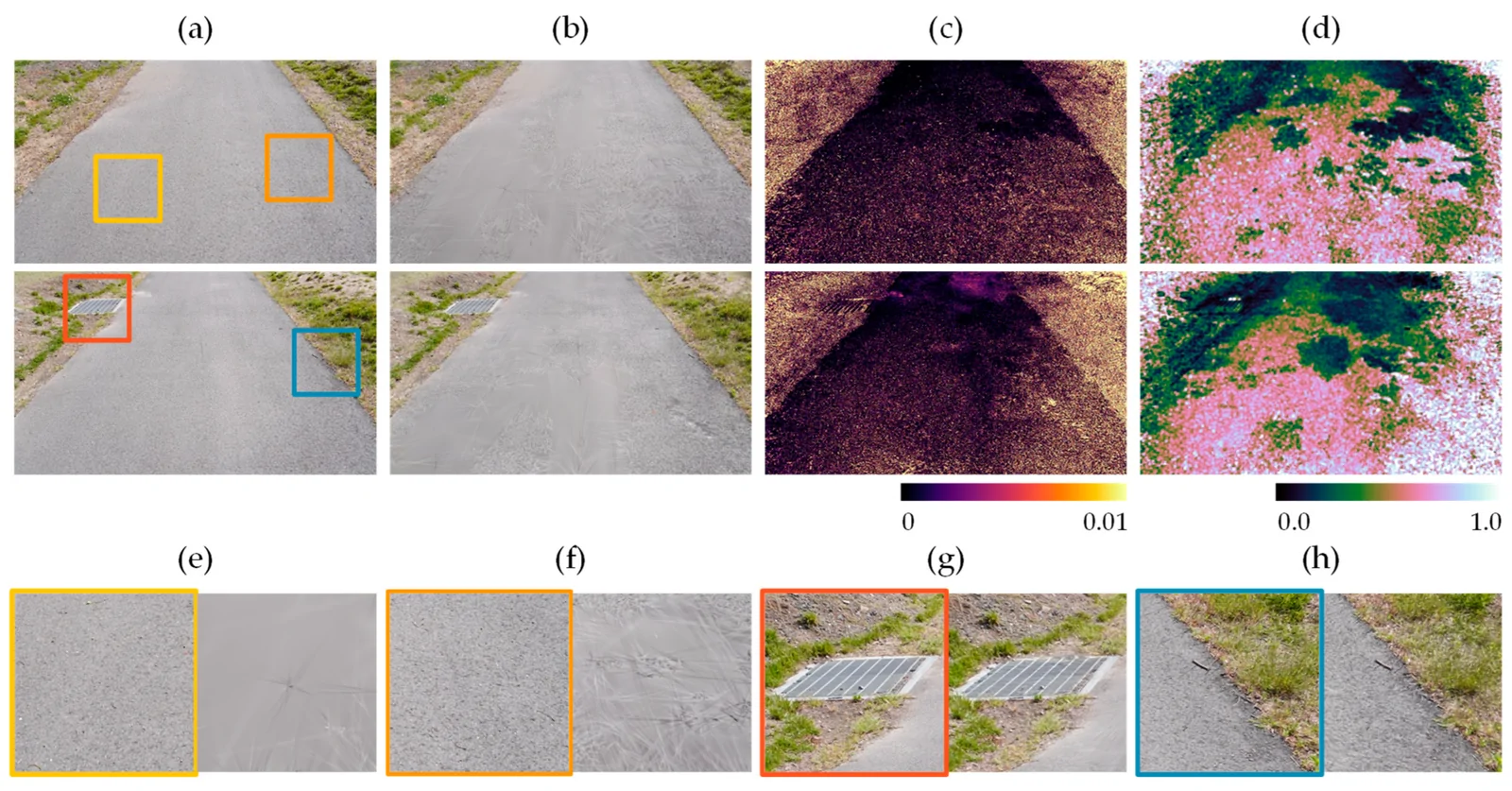
Asphalt-pavement reconstruction results. (**a**) Reference images with the selected ROIs; (**b**) 3DGS-rendered images; (**c**) pixel-wise RGB MSE maps; and (**d**) local SSIM error maps. The color scales range from 0 to 0.01 for MSE and from 0 to 1.0 for local SSIM error. Enlarged reference–rendering comparisons are shown for (**e**,**f**) pavement textures, (**g**) a drainage inlet, and (**h**) the pavement–grass boundary. In each enlarged comparison, the reference image is shown on the left and the corresponding rendering on the right. The colored rectangles indicate the ROIs corresponding to the enlarged comparisons in panels (**e**–**h**).

The asphalt pavement scene focused on a largely planar road surface characterized by low-contrast, repetitive granular texture. The scene also included a drainage inlet, grass, gravel, and the pavement–grass boundary. Because local variations in the pavement texture were small and similar patterns extended over a wide area, this scene provided a challenging case for reproducing fine surface detail. Figure 20 shows the reconstruction results.

The 3DGS rendering reproduced the overall road geometry and general color tone reasonably well. In the enlarged comparisons, the pavement textures in panels (e) and (f) were noticeably blurred and smoothed. The drainage inlet in panel (g), which had clear structural edges, was reconstructed clearly, whereas the pavement–grass boundary in panel (h) remained identifiable but was somewhat blurred.

Over much of the pavement surface, the pixel-wise RGB MSE in panel (c) remained relatively low, whereas the local SSIM error in panel (d) was high, indicating that the overall color tone was preserved but the local textural structure was not. These results indicate that standard 3DGS reproduced clearly defined artificial structures and major scene boundaries reasonably well, whereas low-contrast, repetitive pavement textures were less faithfully preserved. They also demonstrate the importance of evaluating local structural preservation in addition to color-difference-based error.

### 7.5. Sculpture Scene

The sculpture scene represented object-centered outdoor acquisition, in which a relatively isolated target was photographed while the camera moved around it. In this type of acquisition, not only the primary target but also the ground, vegetation, sky, buildings, and other background objects are inevitably included in the images.

**Figure 21:**
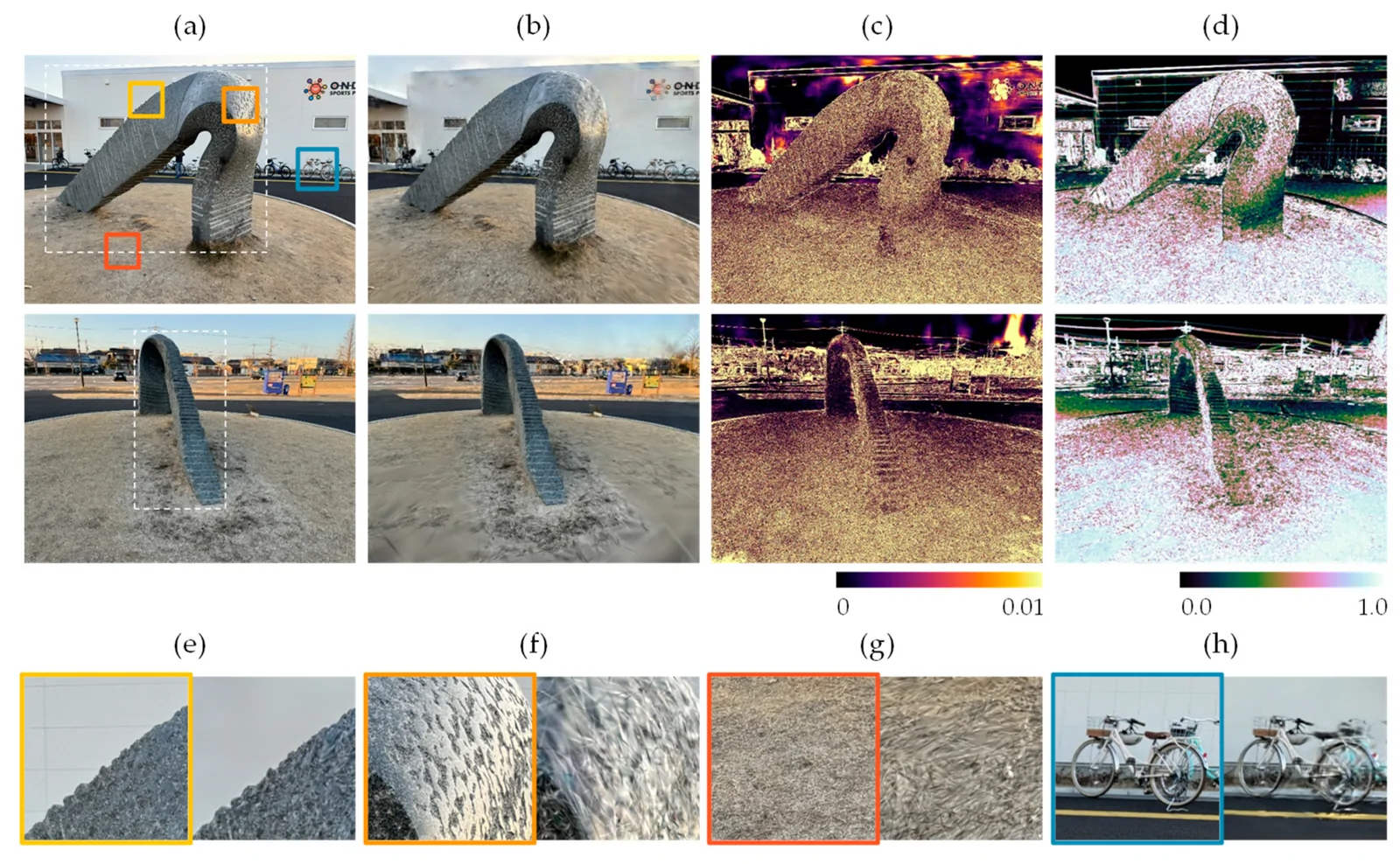
Outdoor-sculpture reconstruction results. (**a**) Reference images with the selected ROIs; (**b**) 3DGS-rendered images; (**c**) pixel-wise RGB MSE maps; and (**d**) local SSIM error maps. The color scales range from 0 to 0.01 for MSE and from 0 to 1.0 for local SSIM error. Enlarged reference–rendering comparisons are shown for (**e**) the sculpture boundary, (**f**) the rough stone surface, (**g**) the ground texture, and (**h**) background bicycles. In each enlarged comparison, the reference image is shown on the left and the corresponding rendering on the right. The white dashed rectangles in the two reference images in (**a**) indicate the fixed foreground bounding boxes used for the target-focused quantitative evaluation. The same bounding boxes were applied to the corresponding reference and rendered images. The colored rectangles indicate the ROIs corresponding to the enlarged comparisons in panels (**e**–**h**).

The target was the outdoor stone sculpture *Twisted Form ’84* by Shiro Hayami, owned by Ube City. The scene included the curved sculpture body, a large central opening, rough stone surfaces, and fine ground textures, together with background objects such as buildings and bicycles located farther from the sculpture. Figure 21 shows the reconstruction results.

The 3DGS rendering clearly reproduced the overall shape of the sculpture and its central opening, while the major contours of the sculpture body were also generally preserved. In the enlarged comparisons, however, although the sculpture edge in panel (e) remained identifiable, the fine surface texture was not sufficiently reproduced. The fine textures of the stone surface in panel (f) and the ground surface in panel (g) were also smoothed or blurred. In addition, the distant bicycles in panel (h) were blurred, and their wheels and other thin components could not be clearly distinguished.

Regarding the background regions, the residential area in the lower test view occupied a relatively large portion of the image and showed large errors in both the pixel-wise RGB MSE map and the local SSIM error map. Although this background region was not the primary reconstruction target in this scene, it may have affected the whole-image quality metrics. By contrast, in part of the white building-wall region, noticeable visual blurring was observed even though the error maps did not necessarily show large values, indicating that local visual degradation may not always be fully reflected in these error maps.

To quantitatively examine the influence of non-target background regions on whole-image evaluation, additional bounding-box evaluations were performed for the two representative test views shown in Figure 21. Fixed rectangular regions enclosing the sculpture, indicated by the white dashed rectangles in Figure 21a, were used for the target-focused evaluation. For the upper view, the whole-image and bounding-box values were 18.7930 and 18.6806 dB for PSNR, 0.4164 and 0.4229 for SSIM, and 0.5618 and 0.5360 for LPIPS, respectively. The differences were therefore relatively small in this view. For the lower view, where the non-target residential background occupied a larger portion of the image and showed relatively large reconstruction errors, restricting the evaluation to the sculpture bounding box increased PSNR from 18.9196 to 19.5772 dB and SSIM from 0.3484 to 0.4016, while reducing LPIPS from 0.6295 to 0.5346. These results indicate that non-target background regions can substantially influence whole-image quality metrics when they occupy a large image area and are reconstructed less accurately. Therefore, in object-centered scenes, whole-image metrics should be complemented by target-focused evaluation when the reconstruction quality of the primary subject is of interest.

Overall, standard 3DGS reproduced the global shape, central opening, and major edges of the sculpture well, whereas the fine textures of the stone surface and ground, as well as small distant background objects, were reproduced less reliably. The bounding-box evaluation further showed that whole-image metrics can be affected by non-target background regions, particularly when such regions occupy a substantial portion of the image. Whole-image metrics should therefore be complemented by target-focused quantitative evaluation and direct ROI-level inspection when the visibility and reconstruction quality of the primary target are important.

### 7.6. Cross-Scene Computational Characteristics

Because camera type, image resolution, image count, scene extent, acquisition trajectory, illumination, and GPU hardware differed among datasets, the computational records in Table 3 are reported only as scene-specific descriptive characteristics and are not used for direct ranking of computational efficiency or scene difficulty. Table 3 summarizes the computational characteristics and test-view quality metrics for each scene and for the different wall-scene training conditions.

For the wall scene, continuing densification until 30,000 iterations under the Densify-30k condition increased the Gaussian count, PLY file size, peak allocated VRAM, and training time compared with the Densify-7k condition. In contrast, the improvements in PSNR, SSIM, and LPIPS for the test views were limited. This result indicates that extending densification and increasing the Gaussian count do not necessarily produce a proportional improvement in image quality.

The forest scene produced the largest model among the evaluated scenes, with 25.1 million Gaussians, a PLY file size of 5.8 GB, and a peak allocated VRAM of 60.8 GB. This scene covered a wide area and contained complex vegetation with abundant fine-scale textures. A relatively large number of images was also used to capture the target area. These characteristics may have contributed to the large Gaussian count, although their individual effects were not evaluated separately. The forest scene was processed on an NVIDIA H100 GPU because its peak allocated VRAM reached 60.8 GB, exceeding the 40 GB memory capacity of the A100 environment available for the other experiments. The H100 was therefore used to accommodate the forest scene at the specified input resolution and training settings. Because the forest training time was not recorded and the GPU hardware differed from that used for the other scenes, its training time was excluded from direct cross-scene comparison.

The Bridge-200 condition achieved the highest test-view quality among the evaluated conditions, with a PSNR of 37.11 dB, an SSIM of 0.952, and an LPIPS of 0.193. In contrast, the asphalt scene contained 9.22 million Gaussians but showed an SSIM of 0.510 and an LPIPS of 0.484, together with smoothing of the low-contrast pavement texture. This contrast indicates that a larger Gaussian count does not necessarily lead to higher test-view quality. The relationship between the high quality metrics obtained under the Bridge-200 condition and the viewpoint configuration of the video-derived images is discussed in the next section.

The remaining scenes exhibited different combinations of model size, computational cost, and image-quality metrics. Overall, neither final Gaussian count nor computational resource consumption alone explained test-view quality. Practical evaluation should therefore consider scene characteristics, local reconstruction quality, and test-view configuration together with model size and computational cost. For the two wall-scene conditions, Gaussian count, PLY file size, training time, and test-view quality metrics in Table 3 are reported as the mean ± standard deviation over three runs.

The quantitative ROI results discussed in Section 7.1, Section 7.2, Section 7.3, Section 7.4 and Section 7.5 are summarized in Table 4.

### 7.7. Effect of Image-Sequence Thinning Under a Fixed Iteration Budget

To investigate the effect of reducing the number of training images in a video-derived image sequence, an additional experiment was conducted using the steel-girder bridge scene. The train/test split and the treatment of camera-pose estimation in COLMAP followed the procedure described in Section 3.2. The same nine test images were retained in all conditions, while the number of training images was varied among 200, 100, and 50. This design enabled the effect of image-sequence thinning on test-view rendering quality to be evaluated while keeping the test views fixed.

**Figure 22:**
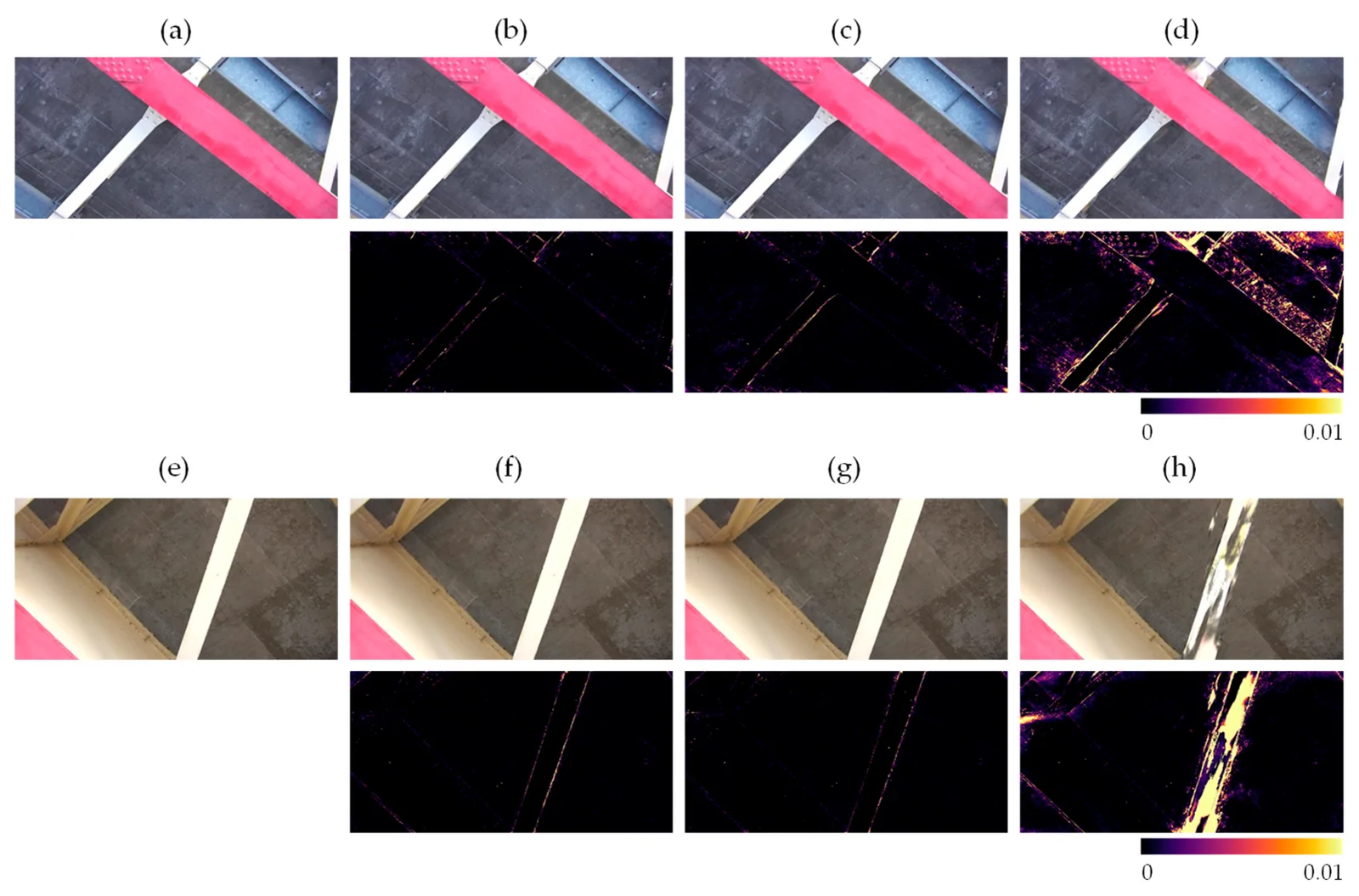
Visual comparison of two full held-out test-view images under different image-sequence-thinning conditions in the steel-girder bridge scene. Panels (**a**) and (**e**) show the reference images; panels (**b**) and (**f**), (**c**) and (**g**), and (**d**) and (**h**) show the corresponding 3DGS renderings obtained using 200, 100, and 50 training images, respectively. The pixel-wise RGB MSE maps are shown below the rendered images using the same color scale from 0 to 0.01. The 200- and 100-image conditions produced visually similar results, whereas the 50-image condition showed increased errors, particularly around the white diagonal member, member boundaries, and fine textures on the underside of the deck.

Figure 22 compares two common full test-view images under the three training conditions, and Table 5 summarizes the corresponding quantitative results. The Bridge-200 and Bridge-100 conditions produced visually similar renderings over the full images. In contrast, the Bridge-50 condition showed more noticeable artifacts and increased MSE, particularly around the white diagonal member, the boundary of the red main girder, and fine textures on the underside of the deck. This visual trend was consistent with the image-level metrics. Reducing the training set from 200 to 100 images caused only a limited decrease in quality, with PSNR decreasing from 37.11 to 36.37 dB, SSIM from 0.952 to 0.947, and LPIPS increasing from 0.193 to 0.202. With 50 training images, PSNR decreased to 31.78 dB and SSIM to 0.919, while LPIPS increased to 0.228.

These results should not be interpreted solely in terms of the absolute number of training images. The steel-girder bridge dataset was extracted from video and covered a relatively limited target area, with the same structural members observed repeatedly from closely spaced viewpoints. The results therefore suggest that sufficient viewpoint coverage may have remained even after the image sequence was thinned. However, the image-overlap ratio was not explicitly measured or controlled in this experiment. Accordingly, the present results do not establish an appropriate or optimal overlap condition, and a systematic evaluation of the relationship between image overlap, rendering quality, and geometric consistency remains a topic for future work.

Regarding computational characteristics, reducing the number of training images did not produce a proportional reduction in final model size or elapsed training time. The final Gaussian counts for the Bridge-200, Bridge-100, and Bridge-50 conditions were 2.82 million, 2.85 million, and 3.09 million, respectively. Thus, the Gaussian count did not decrease with the number of training images and instead increased slightly in the 50-image condition. The PLY file size remained 0.7 GB in all three conditions, while the elapsed training times were also similar at 67 min 49 s, 64 min 58 s, and 65 min 40 s, respectively.

Because the number of optimization iterations was fixed at 30,000, all three conditions performed the same number of single-view rendering, loss-evaluation, and parameter-update steps. The Bridge-200, Bridge-100, and Bridge-50 conditions therefore corresponded to approximately 150, 300, and 600 complete passes through their respective training sets. Consequently, a proportional reduction in elapsed training time with fewer training images was not expected. The similar training times should be interpreted as comparable computational costs under a fixed update budget rather than as evidence that the number of input images has no influence on training cost.

In contrast, peak allocated VRAM decreased from 12.2 GB in the Bridge-200 condition to 9.7 GB and 8.8 GB in the Bridge-100 and Bridge-50 conditions, respectively. Because the final Gaussian count did not decrease, this reduction cannot be explained by Gaussian count alone. The number of input images and the associated image and camera data may also have contributed to peak memory usage in the implementation used in this study.

Overall, reducing the number of training images from 200 to 100 had only a limited effect on test-view quality under the present acquisition conditions, whereas reducing it to 50 produced clearer image-quality degradation. Under the fixed-iteration setting, image-sequence thinning reduced peak VRAM but did not directly reduce final model size or elapsed training time. These findings indicate that the number of images alone is insufficient for evaluating acquisition quality and computational requirements; viewpoint coverage, image overlap, target extent, and the separation between training and test viewpoints should also be considered.

## 8. Discussion

### 8.1. Scene-Dependent Reconstruction Characteristics and Local Error

The cross-scene assessment suggested that reconstruction stability was associated with observation consistency, feature scale, local contrast, and scene and background complexity, rather than being explained by object category alone. Major structures and clearly defined features were generally reproduced well, including the central wall surface, the major structural components of the piled-pier underside, the steel members and selected local details under the Bridge-200 condition, the drainage inlet in the asphalt scene, and the global shape and central opening of the sculpture. In contrast, local degradation remained in sparsely foliated crowns, low-contrast repetitive pavement textures, fine stone and ground textures, the upper wall boundary and regions around the fence, and distant small background objects.

Importantly, reconstruction quality also varied among features within the same scene. In the piled-pier underside scene, rough concrete texture and exposed reinforcement remained clear, whereas leakage stains and discoloration showed different degrees of blurring. Under the Bridge-200 condition, all four selected local features remained identifiable. This within-scene variability reinforces the need for feature-specific ROI evaluation.

The sculpture scene also showed that whole-image metrics and spatial error maps should be interpreted in relation to the primary target. In the lower view, the non-target residential background occupied a relatively large image area and showed large reconstruction errors, and the target-focused evaluation showed that excluding this background substantially changed the image-level metrics. Thus, global metrics and error maps should be complemented by direct ROI-level comparison of the target features. ROI-level verification should be regarded as an application-specific quality-assurance step rather than merely supplementary visual inspection.

The ROI-level metrics did not uniformly correspond to the visual assessment of feature visibility. For example, the pavement-texture ROIs yielded higher SSIM values than the drainage-inlet ROI, although the pavement textures were visibly smoothed and the inlet remained clearly identifiable. Thus, quantitative ROI-level image-similarity metrics should complement, rather than replace, direct visual assessment of the relevant structures and textures.

### 8.2. Quality–Cost Trade-Off and Gaussian/MVS Diagnostics

The wall-scene results demonstrate that rendering quality, computational cost, Gaussian attributes, and MVS-relative geometry capture different aspects of standard 3DGS and should not be treated as interchangeable indicators. Extending adaptive density control from 7000 to 30,000 iterations increased the number of Gaussians, VRAM usage, training time, and PLY file size, whereas the improvement in held-out test-view quality remained modest and the main spatial distribution of local errors changed little. Thus, under the present conditions, the additional quality gain was limited relative to the increase in computational cost.

The number of Gaussians and model size are useful indicators of model scale and resource requirements, but they do not directly explain rendering quality or local-detail preservation. In the wall scene, nearly doubling the final Gaussian count produced only a small improvement in held-out test-view metrics. In the asphalt scene, low-contrast granular textures remained smoothed despite a final model containing more than 9 million Gaussians. Gaussian count, model size, training time, and VRAM usage should therefore be interpreted together with global image-quality metrics and local error distributions.

The bridge image-sequence-thinning experiment further showed that image count, rendering quality, and computational cost were not related in a simple proportional manner. Reducing the number of training images from 200 to 100 had only a limited effect on test-view quality, whereas reducing it to 50 produced clearer degradation. Under the fixed 30,000-iteration budget, thinning reduced peak allocated VRAM but did not reduce the final model size or elapsed training time. These results indicate that image count should be interpreted together with viewpoint coverage, image overlap, and the number of optimization updates when evaluating quality–cost trade-offs.

The Gaussian-attribute analysis showed that Gaussian scales generally decreased during training, although relatively large Gaussians remained in the final model. Large-scale Gaussians were projected more frequently onto the fence and background regions than onto the central wall region. However, the local MSE and local SSIM error distributions substantially overlapped among the four scale–opacity classes, and no clear separation was observed. The scale–opacity classification was therefore insufficient to identify high-error regions or artifacts on its own, although it provided auxiliary information about the spatial characteristics of the Gaussian representation.

The MVS-relative analysis showed that large-scale Gaussians tended to have greater Gaussian–MVS distances than normal-scale Gaussians, although their distributions also substantially overlapped. Median local MSE and local SSIM error generally increased with Gaussian–MVS distance, indicating that greater spatial separation from the MVS-reconstructed wall surface was associated with larger image-space errors in this scene. However, the broad and substantially overlapping percentile ranges showed that Gaussian–MVS distance alone was insufficient to predict local image-space error reliably.

These findings indicate that Gaussian attributes, image-space errors, and MVS-relative spatial agreement are related but provide distinct and complementary information. Because the MVS point cloud was generated from the same image sequence, it is not an independent ground truth and does not establish absolute surveying accuracy. Standard 3DGS should therefore be regarded as a photorealistic visualization layer for representing existing conditions and, where geometric consistency is required, used together with a geometric basis such as MVS or LiDAR point clouds, BIM/CIM, or known dimensions.

### 8.3. Comparison with Existing Studies

Because the target scenes, 3DGS implementations, reference data, and evaluation protocols differ among studies, direct numerical comparison of the reported performance values would not be meaningful. Table 6 therefore compares the scope and primary emphasis of representative studies. Atik [16] combined rendering-quality metrics with M3C2 and cross-sectional comparisons against photogrammetric point clouds for UAV scenes. Fadilah et al. [17] compared the photometric, geometric, and computational performance of multiple 3DGS implementations for UAV-based urban heritage reconstruction. Yan et al. [18] emphasized scalability, geometric accuracy, rendering quality, and computational efficiency in large-scale urban scenes. Fang et al. [29] investigated the effects of image number, resolution, iteration count, and training parameters in indoor scenes, whereas Martin et al. [30] focused on subjective visual quality and the correspondence of objective quality metrics with human perception.

In contrast, the present study uses a common standard 3DGS pipeline to assess rendering quality and computational requirements across six heterogeneous real-world scenes, supplemented by spatial error maps and quantitative and visual evaluation of selected ROIs. Detailed analyses of Gaussian scale–opacity attributes and MVS-relative consistency are conducted for the wall scene. Among the representative studies summarized in Table 6, no single study combines all of these evaluation dimensions. Thus, the contribution of this study is not a new individual metric or 3DGS algorithm, but an integrated practical diagnostic framework that brings these complementary evaluation aspects together and clarifies their different roles and limitations.

### 8.4. Answers to the Research Questions

The results obtained in Section 4, Section 5, Section 6 and Section 7 provide the following answers to the four research questions defined in Section 2.3.

**RQ1:** Under the present experimental conditions, extending adaptive density control from 7000 to 30,000 iterations substantially increased the number of Gaussians, VRAM usage, training time, and model size, whereas the improvement in held-out test-view quality was modest and the spatial distribution of local errors changed little. Thus, continued densification produced diminishing returns in rendering quality relative to its additional computational cost.

**RQ2:** Gaussian scale and learned opacity provided only limited auxiliary information about local image-space errors. Although large-scale Gaussians exhibited different spatial distributions from normal-scale Gaussians, the local MSE and local SSIM-error distributions substantially overlapped among the scale–opacity classes. Therefore, these simple Gaussian attributes alone were insufficient to reliably identify high-error regions.

**RQ3:** Gaussian scale showed an association with MVS-relative distance, and median local image errors generally increased with Gaussian–MVS distance. However, the distributions substantially overlapped, indicating that Gaussian–MVS distance alone could not reliably predict local image-space error. Because the MVS point cloud was reconstructed from the same image sequence, these results represent relative multi-view geometric consistency rather than absolute geometric accuracy.

**RQ4:** Across the six scenes, reconstruction was relatively stable for major structures, clearly defined features, and surfaces observed consistently from multiple viewpoints. In contrast, local artifacts persisted in sparse foliage, low-contrast repetitive textures, fine surface details, object boundaries, and small distant background objects. The cross-scene results therefore suggest that reconstruction stability depends more strongly on observation consistency, feature scale, local contrast, and scene/background complexity than on object category alone.

### 8.5. Practical Implications

The present study did not implement or validate an operational Digital Twin, BIM/CIM overlay, or temporal change-detection workflow. Therefore, the following discussion concerns the evaluation requirements and practical considerations that should be addressed before such integration. The practical suitability of standard 3DGS cannot be judged using a single metric. 3DGS is not a geometric model equivalent to a surveying point cloud or surface mesh, and photorealistic rendering does not guarantee geometric consistency or faithful reproduction of defects. Spatial consistency remains important when a 3DGS model is overlaid with BIM/CIM, point clouds, or inspection records, used to identify defect locations, or compared with models acquired at different times. In temporal comparisons, discrepancies caused by image acquisition, model generation, or registration may otherwise be misinterpreted as actual changes or defect progression. Visual evaluation should therefore be supplemented by consistency checks against an appropriate geometric reference.

Users may also inspect a 3DGS model from arbitrary viewpoints that were not included in the quantitative test-view evaluation. Although such viewpoints do not provide direct reference-image comparisons, they can reveal visual instabilities, occlusion-related artifacts, and regions that were insufficiently observed during image acquisition. Acquisition planning should therefore ensure sufficient image overlap, observation of target surfaces from multiple directions, suitable illumination, and, where feasible, reduced occlusion and unnecessary background coverage.

Care is required when interpreting test-view metrics for densely sampled image sequences. Test frames selected at regular intervals may remain close to adjacent training views, particularly when camera movement is slow or temporarily stops. In such cases, PSNR, SSIM, and LPIPS may primarily measure near-view interpolation rather than generalization to more widely separated viewpoints. The continuous hold-out experiment for the wall scene confirmed this effect: increasing the spatial separation between training and test cameras substantially reduced rendering quality. Therefore, image count, viewpoint spacing, image overlap, and the separation between training and test viewpoints should be reported and interpreted separately. When spatial generalization is important, continuous trajectory hold-out or another split strategy that enforces greater train–test separation should be included in the evaluation.

A practical evaluation may proceed as follows. First, average rendering quality is assessed using PSNR, SSIM, and LPIPS for held-out test views. Second, pixel-wise RGB MSE maps, local SSIM error maps, and magnified ROI comparisons are used to examine the spatial distribution of errors and the visibility of target features. For object-centered scenes, whole-image metrics should also be supplemented with target-masked or ROI-specific metrics where possible. Third, Gaussian count, PLY file size, VRAM usage, and training time are recorded to determine whether the model can be constructed and operated within the available computational environment. Gaussian scale and learned opacity may then be examined as auxiliary descriptors of the internal representation, while recognizing that they do not independently identify artifact regions. Finally, comparison with MVS or LiDAR point clouds, BIM/CIM, known dimensions, or other reference geometry is used to identify major spatial inconsistencies between the visualization layer and the target scene.

### 8.6. Limitations

This study has several limitations. First, the datasets were acquired for different practical purposes, and the camera type, image resolution, number of images, illumination conditions, train–test split, and GPU hardware were not standardized across scenes. The cross-scene results should therefore be interpreted as a descriptive assessment rather than as evidence of causal relationships between individual acquisition factors and reconstruction performance. Accordingly, differences in training time, VRAM usage, and model size across scenes should not be interpreted as effects of scene type alone.

Second, the detailed analyses of Gaussian attributes, local image-space errors, and MVS-relative geometry were conducted primarily for the wall scene. The observed relationships may depend on scene structure, image acquisition geometry, and surface characteristics, and should therefore be validated using additional scenes.

Third, the MVS point cloud was generated from the same image sequence as the 3DGS model and was not an independent ground-truth reference. The Gaussian–MVS analysis consequently assessed relative consistency with an image-based reconstruction rather than absolute geometric accuracy. An independent reference such as LiDAR, TLS, surveyed control, or known dimensions would be required to assess absolute geometric accuracy and to determine how much of the observed distance distribution originates from the 3DGS representation versus the MVS reconstruction itself.

Fourth, the Gaussian-level analyses were based on Gaussian centers, scale, and learned opacity. The Gaussian–MVS distance did not account for Gaussian anisotropy or spatial extent, while the projection-based scale–opacity analysis did not account for view-dependent visibility, projected footprint, depth ordering, accumulated transmittance, overlap among Gaussians, or actual alpha-composited contribution to individual pixels. These analyses should therefore be regarded as exploratory diagnostics of coarse spatial associations rather than contribution-aware error attribution to individual Gaussians.

Fifth, the bridge image-sequence-thinning experiment did not explicitly quantify or control image overlap or viewpoint spacing. In addition, each image set was processed independently using COLMAP. The results should therefore be interpreted as an end-to-end thinning comparison and do not isolate the causal effect of image count or establish an optimal overlap condition. The regular interval-based held-out test-view evaluation can primarily assess interpolation between nearby observed viewpoints, particularly in densely sampled image sequences. To partly address this limitation, an additional continuous spatial hold-out experiment was conducted for the wall scene and showed substantially lower rendering quality when the test viewpoints were more spatially separated from the training cameras. However, this additional evaluation was limited to a single scene and a single continuous hold-out segment. Therefore, the reported results should not be interpreted as a complete assessment of arbitrary-view novel-view synthesis or spatial generalization across all scene types.

Sixth, the image-level quality metrics were calculated over the full test images and therefore included non-target background regions. This was particularly relevant to the object-centered sculpture scene, in which the residential background occupied a substantial image area. To partly address this issue, target-focused bounding-box metrics were additionally evaluated for the two representative sculpture test views shown in Figure 21. However, this evaluation was limited to these representative views and fixed rectangular target regions rather than pixel-level segmentation masks or all test views.

Seventh, run-to-run variability was evaluated using three random seeds for the two principal wall-scene conditions. However, the remaining scene-specific experiments were based on single runs; therefore, their computational and rendering-quality results should still be interpreted descriptively.

Future work should validate the framework under more standardized acquisition conditions and across additional scenes, quantify and control image overlap and viewpoint spacing, perform more systematic target-masked and ROI-specific evaluations across additional object-centered scenes and viewpoints, incorporate independent geometric references such as LiDAR or surveyed control, and develop contribution-aware analyses based on view-dependent rasterization weights.

## 9. Conclusions and Future Work

This study applied standard 3DGS to six real-world image sequences and organized a practical evaluation framework for assessing its potential use as a visualization layer in Digital Twin and inspection-support applications. Standard 3DGS generally reproduced major structures, global shapes, and clearly defined local features well. Local degradation nevertheless remained in sparse foliage, low-contrast or repetitive textures, fine surface details, and distant background objects. In object-centered acquisition, non-target background regions could also influence whole-image quality metrics. These results demonstrate the importance of evaluating global rendering quality together with ROI-level visibility, spatial error distributions, computational requirements, Gaussian attributes, and consistency with reference geometry.

In the wall scene, extending adaptive density control from 7000 to 30,000 iterations substantially increased Gaussian count, VRAM usage, training time, and model size, whereas improvements in held-out test-view quality and local error distributions remained limited. Gaussian scale and learned opacity provided auxiliary information, but the scale–opacity classes did not clearly distinguish high- and low-error regions. Large-scale Gaussians tended to have greater Gaussian–MVS distances, and median local errors generally increased with distance; however, the broad and overlapping distributions showed that distance alone could not reliably predict local image-space error. The bridge thinning experiment similarly showed that image count alone did not determine rendering quality or computational cost. The continuous spatial hold-out experiment further showed that rendering quality decreased substantially when test viewpoints were more spatially separated from the training cameras, highlighting the importance of train–test viewpoint separation when interpreting held-out test-view metrics.

Standard 3DGS should therefore not be treated as a standalone surveying-oriented geometric model. It is better positioned as a photorealistic visualization layer used together with geometric information such as MVS or LiDAR point clouds, BIM/CIM, or known dimensions. The proposed framework provides a structured procedure for determining whether a model provides sufficient visual quality and ROI visibility, can be constructed within available computational resources, and remains spatially consistent with an appropriate geometric reference.

Future work will evaluate image overlap, viewpoint spacing, image count, shooting distance, illumination, and viewpoint coverage under controlled acquisition conditions. More systematic target-masked and ROI-specific evaluations will be investigated across additional object-centered scenes and viewpoints. Contribution-aware Gaussian analysis and temporal comparison methods will also be developed to distinguish actual scene changes from apparent differences caused by model generation, registration, or rendering.

## Figures and Tables

**Table 1:** Target scenes and image acquisition conditions for the cross-scene assessment.

Scene	Scene Characteristics	Data Acquisition	Images/Frames (Total/Train/Test)	3DGS Input Size
Wall	Wall blocks, joints, fence, ground, sky, distant pole	Handheld still images	67/58/9	1500 × 844
Forest	Dense vegetation, road	UAV still images	290/253/37	2640 × 1978
Pier	Dark underside, depth discontinuity	Boat-based video frames	160/140/20	1920 × 1080
Bridge	Shadows, steel members, depth discontinuities	UAV video frames	209/200/9	1920 × 960
Asphalt	Low-texture planar surface	Handheld still images	83/72/11	1500 × 844
Sculpture	Curved object, fine details, low-texture background	Handheld smartphone still images	42/36/6	2822 × 2116

**Table 2:** Gaussian count and mean-scale distribution statistics in approximate real-world units at selected training iterations.

Iteration	Gaussians	Mean [cm]	Median [cm]	P95 [cm]	P99 [cm]
1500	445,114	2.36	1.85	5.60	10.05
3000	1,474,165	1.74	1.40	3.88	7.06
6000	3,850,299	1.33	1.02	3.19	5.26
9000	6,584,283	1.10	0.81	2.83	4.62
15,000	9,023,763	0.92	0.64	2.58	4.34
30,000	8,328,905	0.82	0.53	2.45	4.45

**Table 3:** Computational characteristics and held-out test-view rendering-quality metrics for the evaluated scenes and wall-scene training conditions.

Scene	GPU	SfM Points	Gaussians	PLY Size	Training Time	Peak VRAM	PSNR	SSIM	LPIPS
Wall (Densify-30k)	A100	89,771	8,297,015 (±46,481)	1.916 GB (±0.011)	48 m 10 s (±36 s)	15.3 GB	22.2030 (±0.0547)	0.5482 (±0.0007)	0.2862 (±0.0007)
Wall (Densify-7k)	A100	89,771	4,334,932 (±39,041)	1.000 GB (±0.008)	32 m 33 s (±10 s)	8.3 GB	21.9639 (±0.0404)	0.5422 (±0.0007)	0.3012 (±0.0002)
Forest	H100	251,035	25,105,142	5.8 GB	N/A	60.8 GB	24.0864	0.7516	0.2737
Pier	A100	76,296	1,756,118	0.4 GB	34 m 39 s	8.6 GB	27.5804	0.7948	0.5512
Bridge-200	A100	153,724	2,817,939	0.7 GB	67 m 49 s	12.2 GB	37.1128	0.9524	0.1933
Asphalt	A100	39,122	9,218,661	2.1 GB	61 m 56 s	16.1 GB	23.9367	0.5098	0.4841
Sculpture	A100	17,463	1,619,913	0.4 GB	81 m 55 s	8.9 GB	18.0944	0.3963	0.4844

N/A indicates that training time was not recorded for the forest scene. Values in parentheses for the two Wall conditions indicate the standard deviation over three runs.

**Table 4:** Quantitative evaluation of selected regions of interest (ROIs). Colored squares correspond to the ROI rectangles shown in the reference images in Figure 17, Figure 18, Figure 19, Figure 20 and Figure 21.

Scene	ROI	ROI Size (px)	PSNR (dB)	SSIM	LPIPS
Forest	■ Sugi crown	270 × 270	24.9566	0.7686	0.1869
	■ Hinoki crown	270 × 270	22.2010	0.7195	0.2116
	■ Red pine crown	270 × 270	20.1503	0.3694	0.3895
	■ Road boundary	270 × 270	27.2731	0.8672	0.1739
Pier	■ Leakage stain	240 × 240	35.0254	0.8666	0.3728
	■ Surface discoloration	240 × 240	30.0142	0.8386	0.5538
	■ Rough concrete texture	240 × 240	29.9842	0.8560	0.2289
	■ Exposed reinforcement	240 × 240	33.6033	0.9050	0.2461
Bridge	■ Fastener/member boundary	420 × 420	35.3177	0.9529	0.1720
	■ Coating deterioration	420 × 420	38.3716	0.9508	0.1946
	■ Overlapping connection	420 × 420	37.5901	0.9545	0.2285
	■ Small spot-like feature	420 × 420	34.6846	0.9557	0.1837
Asphalt	■ Pavement texture 1	270 × 270	29.1457	0.7866	0.1291
	■ Pavement texture 2	270 × 270	28.4743	0.8597	0.1179
	■ Drainage inlet	270 × 270	23.7712	0.7111	0.2051
	■ Pavement–grass boundary	270 × 270	19.9432	0.3454	0.3308
Sculpture	■ Sculpture boundary	290 × 290	21.0694	0.5461	0.4809
	■ Rough stone surface	290 × 290	15.0898	0.1632	0.6047
	■ Ground texture	290 × 290	17.6258	0.1404	0.6293
	■ Background bicycles	345 × 345	18.7774	0.6760	0.4527

**Table 5:** Effect of image-sequence thinning under a fixed iteration budget in the steel-girder bridge scene.

Condition	COLMAP Images	Train	Test	SfM Points	Gaussians	PLY Size	Training Time	Peak VRAM	PSNR	SSIM	LPIPS
Bridge-200	209	200	9	153,724	2,817,939	0.7 GB	67 m 49 s	12.2 GB	37.1128	0.9524	0.1933
Bridge-100	109	100	9	84,672	2,854,183	0.7 GB	64 m 58 s	9.7 GB	36.3650	0.9467	0.2017
Bridge-50	59	50	9	47,772	3,089,245	0.7 GB	65 m 40 s	8.8 GB	31.7796	0.9190	0.2280

**Table 6:** Comparison of representative studies according to scene scope and evaluation dimensions for 3D Gaussian Splatting.

Study	Scene	3DGS Method	Rendering/ Local Evaluation	Geometry/Internal Analysis/Computation	Main Contribution
Atik [16]	UAV scenes	Splatfacto (vs. NeRF)	Rendering-quality metrics	M3C2 and cross-sectional analysis vs. photogrammetric point clouds; training/testing time	NeRF–3DGS comparison for UAV point-cloud generation
Fadilah et al. [17]	UAV urban heritage	Native 3DGS; PostShot Splat3; MCMC variants (PS and LFS)	Photometric metrics; qualitative artifact assessment	MVS-based geometry; training time; Gaussian count	Comparison of photometric, geometric, and computational performance
Yan et al. [18]	Large-scale urban scenes	Multiple GS methods	Rendering quality	Geometric accuracy, scalability, computational efficiency	Comprehensive evaluation for urban reconstruction
Fang et al. [29]	Indoor scenes	Standard 3DGS with parameter variations	Rendering-quality metrics	Training and rendering time	Parameter effects and optimization for indoor scenes
Martin et al. [30]	Multiple visual scenes	Multiple GS methods	Objective metrics and subjective quality assessment	Training and synthesis time; model size	Subjective quality benchmark and evaluation of objective metrics
Ours	Six real-world scenes	Standard 3DGS	Global metrics; spatial error maps; quantitative ROI evaluation	Gaussian-attribute analysis; MVS-relative consistency; Gaussian count, VRAM, training time, and model size	Integrated practical diagnostic evaluation across heterogeneous real-world scenes

## Data Availability

The wall and asphalt image datasets are available from the corresponding author upon reasonable request. The forest, piled-pier underside, and steel-girder bridge datasets are not publicly available because they were provided by third parties and are subject to collaborative restrictions. The sculpture dataset is not publicly available because its use is subject to permission from Ube City.
